# O-GlcNAcylation-dependent liquid-liquid phase separation regulates the nuclear translocation of YAP to exacerbate vascular neointimal hyperplasia

**DOI:** 10.7150/thno.113303

**Published:** 2025-07-11

**Authors:** Ping Weng, Yilin Wen, Zhiyi Yuan, Limei Ma, Liming Yang, Chengju Li, Wanping Zhang, Chao Yu

**Affiliations:** 1 College of Pharmacy, Chongqing Medical University, Chongqing, 400016, China.; 2 Chongqing Key Laboratory for Pharmaceutical Metabolism Research, Chongqing,400016, China.

**Keywords:** vascular neointima hyperplasia, yap, O-GlcNAcylation, liquid-liquid phase separation, hesperidin

## Abstract

**Background:** Suppressing the abnormal proliferation and migration of vascular smooth muscle cells (VSMCs), key pathological features of vascular neointimal hyperplasia (NIH), is an effective strategy for treating vascular insufficiency disorders caused by intimal remodeling. Increasing evidence suggests that Yes-associated protein (YAP) contributes to the abnormal proliferation and migration of VSMCs. However, the mechanisms by which YAP leads to NIH are poorly understood.

**Methods:** An Immunofluorescence assay was used to detect the expression and distribution of YAP in mice or rats induced by ligation or balloon injury of the carotid artery. LC/MS, Co-immunoprecipitation (Co-IP), and confocal microscopy were used to evaluate O-GlcNAcylation, nucleation, and liquid-liquid phase separation (LLPS) of YAP, respectively. Protein-Protein Interaction Network (PPI) was used to predict potential binding substrates for YAP. The fluorescence recovery after photobleaching (FRAP) was applied to detect the binding of YAP to the substrate. Multiple biochemical analyses were conducted to unravel the underlying mechanisms.

**Results:** YAP expression in synthetic-type VSMCs was highly increased in the injured artery. The up-regulated YAP in the nucleus of VSMCs increased transcription of the target gene CYR61. Knockdown of YAP and mutation of YAP O-GlcNAcylation sites in VSMCs *in vitro* attenuated PDGF-BB-induced abnormal proliferation and migration. This process was primarily due to the reduction of O-GlcNAcylation of YAP, which led to decreased LLPS of YAP and subsequently reduced the combination of YAP with the nuclear protein STAT3. Consequently, the nuclear translocation of YAP was affected, ultimately impacting the mRNA levels of CYR61, PCNA, OPN, and α-SMA. The small molecule hesperidin could inhibit YAP nuclear translocation and suppress NIH.

**Conclusion:** Our findings revealed that O-GlcNAcylation-dependent LLPS regulates the nuclear translocation of YAP as a critical mechanism promoting NIH progression and may provide new strategies to prevent NIH.

## Introduction

Carotid artery stenosis is one of the critical causes of cerebral ischemia, and 70-89% of carotid artery stenosis is related to previous transient ischemic attacks 1-4. The findings suggest that neointima formation is a pathological hallmark of stenosis, and the leading causes of its formation include the abnormal proliferation and migration of vascular smooth muscle cells (VSMCs) and the buildup of extracellular matrix 5,6. Therefore, inhibiting the abnormal migration and proliferation of VSMCs is important for treating vascular-related diseases. In the early stage of neointima formation, endothelial injury stimulates the conversion of VSMCs from contractile to synthetic types, further promoting the migration of VSMCs into the subendothelial space and their self-proliferation, ultimately leading to the development of stenosis. Recent studies suggest that the promotion of VSMC proliferation and migration may be linked to the regulation of multiple factors, including the control of Bach1 protein 7. This phenomenon might involve Glut10-mediated SMC mtDNA demethylation as a genetic modification and P4HA2-induced YAP1 prolyl hydroxylation as a post-translational modification, and that inhibition might involve Glut10-mediated SMC mtDNA demethylation as a genetic modification 8,9. However, the precise mechanisms governing the regulation of VSMC proliferation and migration remain elusive.

Research on VSMC proliferation and migration has gradually focused on gene transcription, protein translation, and post-translational modifications 10-14. Among them, the Yes-associated protein (YAP), a potent regulator of the Hippo pathway, has been identified for its promotional effect on the proliferation and migration of VSMCs 15,16. Phosphorylation of YAP leads to its retention in the cytoplasm and subsequent ubiquitination and degradation. However, YAP translocates to the nucleus in a non-phosphorylated state and regulates the expression of downstream pro-proliferation and migration genes by binding to transcription factors 17. O-GlcNAcylation of YAP has been shown to competitively inhibit its phosphorylation modification, affecting protein localization and function 18-20. However, the molecular mechanism of O-GlcNAcylation remains unclear. It is a post-translational modification that attaches O-linked β-N-acetylglucosamine (O-GlcNAc) moieties to either serine or threonine residues of proteins, mainly through the O-GlcNAc transferase OGT. Subsequently, O-GlcNAc hydrolase (OGA) de-O-GlcNAcylates the protein. O-GlcNAcylation by OGT utilizes uridine diphosphate N-acetylglucosamine (UDP-GlcNAc) as a precursor molecule synthesized by the hexosamine biosynthesis pathway (HBP). Current studies have shown that O-GlcNAc modification is crucial in regulating protein structure, cellular localization, and biological functions 21-24.

YAP is transported from the cytoplasm to the nucleus to activate the transcription of target genes. This process involves a molecular dynamic mechanism for the compartmentalized distribution of proteins in the cell 25-28. Recent studies have found that O-GlcNAc modification can regulate the membrane-less compartmentalized assembly of proteins through the strong hydrophilicity and multivalent interactions of sugar molecules, thereby affecting the formation of protein aggregates 29-31. YAP forms liquid condensates within the nucleus that sequester the YAP transcription factor TEAD1 and other YAP-associated coactivators, including TAZ, and subsequently induce the transcription of YAP-specific proliferation genes 22,27,32. Protein aggregates can be regarded as a membrane-less organelle with “high reactant concentration”, one of the critical mechanisms that can dynamically, rapidly, and reversibly regulate protein aggregation and dispersion 33-37.

Moreover, post-translational modification influences the aggregation of YAP 38-41. We found multiple O-GlcNAc sites in the Intrinsically Disordered Regions (IDRs) of the YAP protein, which become ordered during the phase separation process, providing structural features that can be exploited for the biological function. Therefore, we speculated that O-GlcNAc modification may be involved in the formation of YAP protein aggregates and thus regulate its spatial translocation. However, YAP lacks a classical nuclear localization signal, and its entry into the nucleus may require other nuclear translocator proteins 42-44. A recent study reported the co-localization of YAP with STAT3 in the endothelial cytoplasm and nucleus of tumor-associated ECs, suggesting that the STAT3 nuclear shuttling mechanism is required for the nuclear translocation of YAP 45. STAT3 is a member of the signal transducer and activator of the STAT family, which participates in the proliferation of VSMCs 46.

In this study, we demonstrated that the O-GlcNAc modification of YAP increased during vascular stenosis. This change promoted the binding of YAP to STAT3 by affecting the formation of cytoplasmic YAP protein condensate and translocating it to the nucleus. Ultimately, YAP exerted its pro-proliferative and migratory role in the nuclei of VSMCs with the help of STAT3. By intervening in the O-GlcNAc modification of YAP to reduce its nuclear translocation, the neointima formation can be effectively slowed down by using small-molecule natural drugs, providing a new idea for treating carotid stenosis-type vascular diseases in the clinic.

## Methods

**Animal:** All mice and rats were maintained in the Center for Laboratory Animals of Chongqing Medical University (Chongqing, China). All manipulations involving live mice were carried out according to established ethical guidelines and with the approval of the Animal Care & Welfare Committee at Chongqing Medical University (approval numbers IACUC-CQMU-2023-0275).

**Mouse primary aortic smooth muscle cell extraction:** Mouse thoracic aortic smooth muscle cells were extracted according to existing methods 47, and 2-10 generations of stable primary cells were taken for experiments. DMEM/F12 medium was purchased from Gibco, fetal bovine serum (FBS) was purchased from Shanghai Taitan, and penicillin-streptomycin and phosphate buffer were purchased from Beyotime.

**Balloon injury of the left common carotid artery in rats:** After isoflurane respiratory anesthesia using an anesthesia ventilator for SD rats, the bifurcation of the left common carotid artery was exposed, and the left common carotid artery, left internal carotid artery and left external carotid artery were isolated, the left external carotid artery was ligated, the proximal end of the left common carotid artery and the left internal carotid artery were clipped with arterial clips, and the left external carotid artery was clipped with ophthalmic forceps, and the small port of the left external carotid artery was cut, and a balloon inserted, and inflated, and the arterial clips of the left common carotid artery opened, and then repeatedly pumping with the balloon inflated three times. After treatment, the left external carotid artery wound was ligated, and the arterial clips at the left internal carotid artery and the left common carotid artery were removed to restore the blood flow supply as the injury group; the right common carotid artery was isolated without ligature and balloon injury, and the rest was consistent with the left side as the sham operation group; the left and right common carotid arteries were harvested at the modelling site of the SD rats after the wound was closed and waited for 14 days.

**Mouse left common carotid artery ligation model:** After isoflurane respiratory anesthesia using an aesthetic ventilator, C57BL/6 mice were exposed to the bifurcation of the left common carotid artery, the left common carotid artery was isolated and ligated near the bifurcation, and the operation was completed. The sham-operated group was divided into the operated group + lysate group, the operated group + low-dose 25 mg/kg hesperidin group, the operated group + high-dose 50 mg/kg hesperidin group, the sham-operated group + vehicle group, the sham-operated group + high-dose 50 mg/kg hesperidin group. The sham-operated group was divided into the surgery group + vehicle group, surgery group + low dose 25 mg/kg hesperidin group, surgery group + high dose 50 mg/kg hesperidin group, sham-operated group + vehicle group, and sham-operated group + high dose 50mg/kg hesperidin group.

**HE staining:** Rat and mouse carotid arteries were fixed with 4 % formalin and paraffin-embedded. Routine hematoxylin/eosin staining was used to assess endothelial hyperplasia, respectively, as described previously 48.

**Immunofluorescence of vascular paraffin sections:** Vascular paraffin sections were placed in a 60°C oven for 1.5 h, immersed in xylene for 30 min (continued in 60°C), taken out of the oven, washed 3 times with PBS for 5 min each time, immersed in 1X sodium citrate solution, microwaved on high until slightly boiling and then turned to low for 10 min, cooled the sections, washed in PBS for 5 min, and incubated with 5% BSA + 0.3% Triton 100X The sections were closed at room temperature or 37°C for 1 h, incubated with primary antibody at 4°C overnight or 37°C for 4 h, washed 3 times with PBST for 5 min each time, incubated with Alexa Fluor 488 or fluorescent secondary antibody labeled with Alexa Fluor 555 at 37°C for 1 h at room temperature and protected from light, and washed with PBST 3 times for 5 min each time, and incubated with DAPI at room temperature and protected from light for 10 min, and washed with PBST for 5 min and then sealed. DAPI was incubated for 10 min at room temperature, washed with PBST for 5 min, and then the slices were blocked. The corresponding primary antibodies were YAP (proteintech, 13584-1-AP, 1:200), Alpha-SMA (Zen-bio, 1:200), OPN (proteintech, 22952-1-AP, 1:200), HIC5 (proteintech, 10565-1-AP, 1:200), OGT (Zen-bio, R25212, 1:200), GLUT1 (Zen-bio, 380464, 1:200), STAT3 (Cell Signaling, #9139, 1:200).

**Cellular immunofluorescence:** Cells were inoculated in 24-well plates containing 9×9 cm slides, washed three times with PBS, fixed with 4% paraformaldehyde for 10 min at room temperature, and washed three times with PBS for 5 min each time; the plate was closed with 5% BSA+0.3% Triton 100X mixing closure solution for 1 h at room temperature, then added with primary antibody dilution and incubated at 4 °C overnight, and washed with PBS three times for 5 min each time; the plate was sealed after incubation with Alexa Fluor 488 and Alexa Fluor 555 labeled fluorescent secondary antibody for 1 h at room temperature and protected from light, and then washed with PBS for 5 min each. Alexa Fluor 488 and Alexa Fluor 555 are labelled fluorescent. Secondary antibodies were incubated for 1 h at room temperature protected from light, and washed with PBS three times, each time for 5 min.

**Western blotting analysis:** For western blotting (WB), the proteins were resolved on SDS-polyacrylamide gel electrophoresis (SDS-PAGE) gels followed by standard WB. The first resistance has beta-Actin (proteintech, 81115-1-RR, 1:5000), YAP (proteintech, 13584-1-AP, 1:1000), Alpha-SMA (Zen-bio, 1:1000), OPN (proteintech, 22952-1-AP, 1:1000), STAT3 (Cell Signaling, #9139, 1:1000), O-GlcNAc (Santa Cruz, sc-59623, 1:1000).

**Quantitative Real-Time PCR (qRT-PCR):** Relative quantitative RT-PCR (qPCR) was carried out using SYBR premix Ex Taq (AG). The mouse specific gene primers are as follows: β-Actin: F-CCACAGCTGAGAGGGAAATC, R-AAGGAAGGCTGGAAAAGAGC; YAP: F-ACCCTCGTTTTGCCATGAAC, R-TTGTTTCAACCGCAGTCTCTC; CYR61: F-GCGCTAAACAACTCAACGA, R-TTCACAGGGTCTGCCTTC.

**Cell counting:** Cells were grown in 12-well plates for 24 h after serum-free starvation for 12 h. The control group was added serum-free DMEM medium, and the experimental group was treated with 10 ng/ml PDGF-BB (bioworld, BK0152) for 24 h. The cells were washed once with PBS, digested with trypsin, and resuspended by adding 500 μL of medium and counted by using the cell counting plate.

**Cell Counting Kit-8 (CCK8):** Cells were inoculated in 96-well plates for 24 h after serum-free starvation for 12 h. The control group was added serum-free DMEM medium, and the experimental group was treated with 10 ng/ml PDGF-BB for 24 h. The medium was discarded, washed twice with PBS, and 100 μL of CCK8 working solution with 10% concentration was added, and absorbance (OD) was measured using an enzyme labeling instrument (Instruments, Inc.) under the condition of 450 nm—value measurement.

**Wound healing experiment:** Cells were inoculated in 12-well plates for 24 h. When the cells grew to about 90% density, they were serum-free and treated for 12 hours. Lines were drawn with a 200 μL lance tip and washed twice with PBS, serum-free DMEM medium was added to the control group, and the experimental group was treated with 10 ng/ml PDGF-BB for 24 h. The cells were photographed under a Nikon optical microscope.

**Transwell assay:** Transwell assays were conducted in 24-well plates. Cells were plated in Transwell for 24 h. Serum-free medium was added to the upper and lower chambers of the control group, and serum-free medium was added to the upper chamber and 10 ng/ml PDGF-BB to the lower chamber of the experimental group; the cells were cultured for 24 h. The cells were washed once in PBS, fixed in 4% paraformaldehyde for 30 min, and then washed once in PBS, and then stained in 1% crystal violet for 1 h. The cells were gently swabbed in the upper chamber and the migrated cells in the lower chamber by Nikon orthogonal light microscope. The cells in the upper chamber were gently swabbed with a cotton swab, and the migrated cells in the lower chamber were observed with a Nikon orthogonal light microscope.

**Liquid Chromatograph Mass Spectrometer (LC/MS):** 3×10^6^ MASMC that were untreated or stimulated with PDGF-BB for 24 h were harvested and washed twice with PBS. Cells were digested with trypsin, cell suspension was collected and washed and resuspended in PBS buffer. Cells were collected by centrifugation at 1000 g for 5 min at room temperature to remove the PBS supernatant. The cells were resuspended by adding 100 μL of mobile phase A+B mixture, and the cells were fully broken by an ultrasonic cell crusher with a programmed of 30% power, 3 times in 3 seconds, with an interval of 3 seconds each time. The broken cell suspension was centrifuged at 13000 rpm for 10 min at 4°C. 100 μL of the supernatant was taken, 400 μL of methylene chloride was added, and the cells were shaken at room temperature for 10 min and centrifuged at 13000 rpm for 10 min. The upper aqueous phase was taken and diluted 10-fold with the mobile phase A + B mixture, and the supernatant was extracted using the Multiplexed Reaction Monitoring (MRM) system with the negative mode of an Agilent G6470A Liquid Chromatography Mass Spectrometer-reaction monitoring (MRM) LC-MS method on an Agilent G6470A liquid chromatography-mass spectrometer for relevant metabolite analysis. Peak areas integrated using MassLynx4.1 were normalized to the corresponding metabolite concentrations. The liquid chromatographic conditions for the LC-MS assay described were as follows: the chromatographic column was an ACQUITY UPLC HSS T3 column with a size of 2.1 150 mm and pore size of 1.8 um; the mobile phase A was a 5% aqueous acetonitrile solution containing 0.5 mM ammonium acetate and 1.5 mM DBAA, and the mobile phase B was an 85% aqueous acetonitrile solution containing 0.5 mM ammonium acetate and 6 mM DBAA.

**Co-immunoprecipitation (Co-IP):** The Protein A/G Magnetic Beads used were purchased from MCE (HY-K0202A) and the experiments were performed according to their recommended methods. Antibodies: YAP (proteintech, 13584-1-AP, 1:100), STAT3 (Cell Signaling, #9139, 1:1000). O-GlcNAc (Santa Cruz, sc-59623, 1:1000).

**Actinomycin ketone assay:** Cells were inoculated in 12-well plates, and after 24h of adherent cell growth, the cells were treated with DMSO, 15 μM OSMI-1 (MCE, HY-119738), 10 μM TMG (MCE, HY-12588) for 24 h, and 20 μM CHX for another 0, 0.5, 1, 2 and 4 h, respectively, and then the samples were collected and subjected to WB for detection of YAP protein expression.

**siRNA interference, plasmid transfection:** Cells were inoculated in 12-well plates to reach a density of about 50-60%, and the exchange solution was a fresh, non-resistant medium. siRNA or plasmid dilutions were mixed with transfection reagent dilutions and left to stand for 20 min, then the mixture was added dropwise to the well plates, and gently shaken from side to side to make it uniformly distributed in the well plates, and the incubation was continued for 24-48 h for the subsequent operations.

**Photobleaching fluorescence recovery experiment:** Cells were inoculated in a special dish for fluorescence confocal microscopy and transfected with fluorescently labelled plasmids after 12 h. After 18 h, the cells were dosed as required for the experiment. Turn on the heat preservation measures during confocal microscope observation. For droplets formed by phase separation, RFP photobleaching of selected droplets was generally performed with 80% laser intensity and the recovery time was recorded. The bleached fluorescence intensity was normalized to 100% and an average fluorescence intensity recovery curve was produced.

**Thermal stability test:** Tests were performed according to previously reported methods, and MASMC inoculated into 10 cm dishes were cultured at about 90% density, and cells were collected from 2 × 10 cm dishes. Cells were first washed twice with PBS, plus 1 mL of PBS + PI (100×) protease inhibitor scraped off the cells, lysed on ice for 30 min, then thawed in liquid nitrogen for 5-10 s, 37°C water bath, repeated three times, and centrifuged at 20,000 g, 4°C, for 20 min, and the supernatant was collected for protein quantification. The supernatants were divided into two parts, one with DMSO as control and one with 100 μM of hesperidin, and the reaction was shaken at room temperature for 1-3 h. After the reaction, each group was divided into ten equal parts, which were added to the PCR tubes and labeled well, and the PCR machine was set up in advance, and the tubes were put into the machine according to corresponding temperatures to carry out the reaction of different temperature gradients (the difference of temperatures was set at 5°C, starting from 37°C). At the end of the reaction, centrifuge at 20,000g for 20 min at 4°C, collect an equal amount of supernatant into a 1.5 mL EP tube, add the appropriate concentration volume of Loading buffer, and cook the sample at 95°C for 6 min. The rest of the steps are the same as WB, perform SDS-PAGE gel separation (10% concentration of the separator gel), transfer the membrane, 5% BSA closure, YAP antibody incubation primary antibody, secondary antibody incubation, and ECL after incubation. The primary antibody was incubated with a YAP antibody, and the secondary antibody was incubated and developed by ECL. The experiment was to be repeated at least three times. The intensity of the bands was quantified by Image J software and the fitted curves were calculated by Origin2021 data software 49**.**

**Calculation method of p-YAP/YAP:** After acquiring images using a chemiluminescence imager, we utilized ImageJ to quantify the gray values. Both p-YAP and total YAP were normalized to the gray value of Actin, allowing us to determine their relative protein expression levels in relation to Actin. Subsequently, by comparing the normalized values of p-YAP with those of total YAP, we obtained the relative phosphorylation level of YAP for each experimental group.

**Statistical Analysis:** Statistical analyses and graphs were performed by using Prism (GraphPad 9.0 Software). Statistical significance was determined by a two-tailed, unpaired Student's t-test (for two groups comparing) or one-way ANOVA with Tukey post-hoc test (for more than two groups comparing). Statistical data were presented as mean ± standard error of the mean ± SEM. p <0.05 was considered statistically significant. *p <0.05; **p <0.01; ***p <0.001; ****p < 0.0001. Data were independent biological replicates. All quantifications and statistics have already been documented in the figure legend.

## Results

### YAP promotes vascular neointima formation by increasing VSMC proliferation and migration

**Figure 1:**
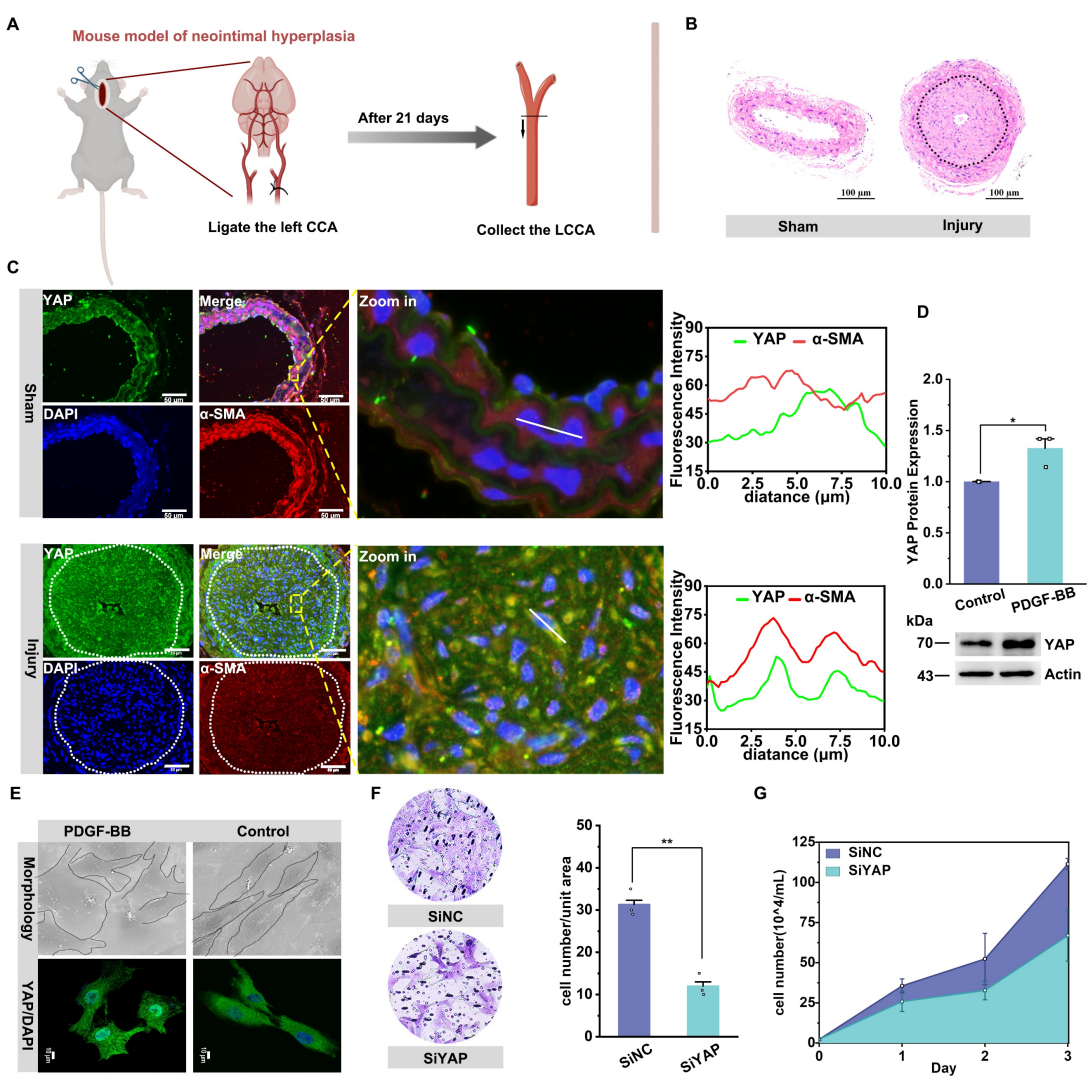
** YAP promotes the proliferation and migration of VSMCs in vascular neointima hyperplasia.** A) Schematic diagram of the experimental design. Adult mice (6-8 weeks of age) were randomly divided into two groups to receive left carotid ligation and sham operation, respectively. After 21 days, B) HE staining was performed on the left carotid artery from the ligation site to the proximal end to observe neointima formation. C) Confocal imaging of YAP (green) and smooth muscle cell marker α-SMA (red) co-localization in blood vessels (n = 3). Areas outlined by rectangles in the merged images are enlarged at right. Scale bars, 50 µm. The linear co-localization plots of YAP and α-SMA outlined by horizontal lines in the enlarged image are shown on the right. D) Immunoblot analysis and quantitative maps of YAP in Primary mouse aortic smooth muscle cells (PMASMCs) treated with or without PDGF-BB (10 ng/mL) for 24 h (n = 3). *p < 0.05 (unpaired, two-tailed Student's test). E) Scanning electron microscope (SEM) images of PMASMCs treated with or without PDGF-BB and immunofluorescence images of protein YAP (n = 3). F) The Transwell Migration Assay image and the quantitative number of migrating cells after siYAP silencing PMASMCs indicate the migration ability of the cells and G) the cell count indicates the proliferation of the cells. **p < 0.01 (unpaired, two-tailed Student's test).

We constructed a rat balloon injury model and a mouse carotid artery ligation model to verify the role of YAP on cell proliferation and migration, respectively, in vascular neointima formation (**Figure 1A**). We found that mice in the surgery group had obvious neointima formation on the inner side of the carotid artery (Figure 1B, S1A). The neointima was mainly composed of synthetic-type vascular smooth muscle cells (VSMCs), as determined by α-SMA and OPN immunostaining (Figure S1A), and exhibited high YAP expression (Figure 1C; Figure S1B). Subsequently, in an in vitro model of proliferation and migration of VSMCs induced with PDGF-BB (Figure S1C & D), a significant elevation of the synthetic-type marker OPN was observed (Figure S1I). Meanwhile, YAP was similarly highly expressed in synthetic-type VSMCs, accompanied by a change in cell morphology from the original shuttle shape to an irregular polygon (Figure 1D & E). Notably, CYR61 (CCN1), a pivotal downstream effector of the YAP/TEAD transcriptional complex in the Hippo pathway, was transcriptionally activated via direct binding of nuclear YAP/TEAD to conserved promoter TREs 50. With increasing levels of YAP in the nucleus, YAP-driven CYR61 promotes VSMCs phenotypic switching, proliferation, and neointimal hyperplasia. *In vivo* studies also demonstrated CYR61-YAP co-upregulation in vascular injury models 51-53. We observed increased transcription of the downstream target gene CYR61 (Figure S1J). Further, we knocked down YAP with siRNA and found that the proliferation and migration of VSMCs were significantly diminished (Figure 1F & G, Figure S1E & F). In contrast, transfection of plasmids overexpressing YAP enhanced the proliferation and migration of VSMCs (Figure S1G & H). The results of the in vivo and in vitro experiments jointly indicated that YAP played a facilitating role in the proliferation and migration of VSMCs to participate in the formation of neointima.

### O-GlcNAcylation enhances YAP stability and nuclear translocation to promote YAP-driven VSMC proliferation and migration

**Figure 2:**
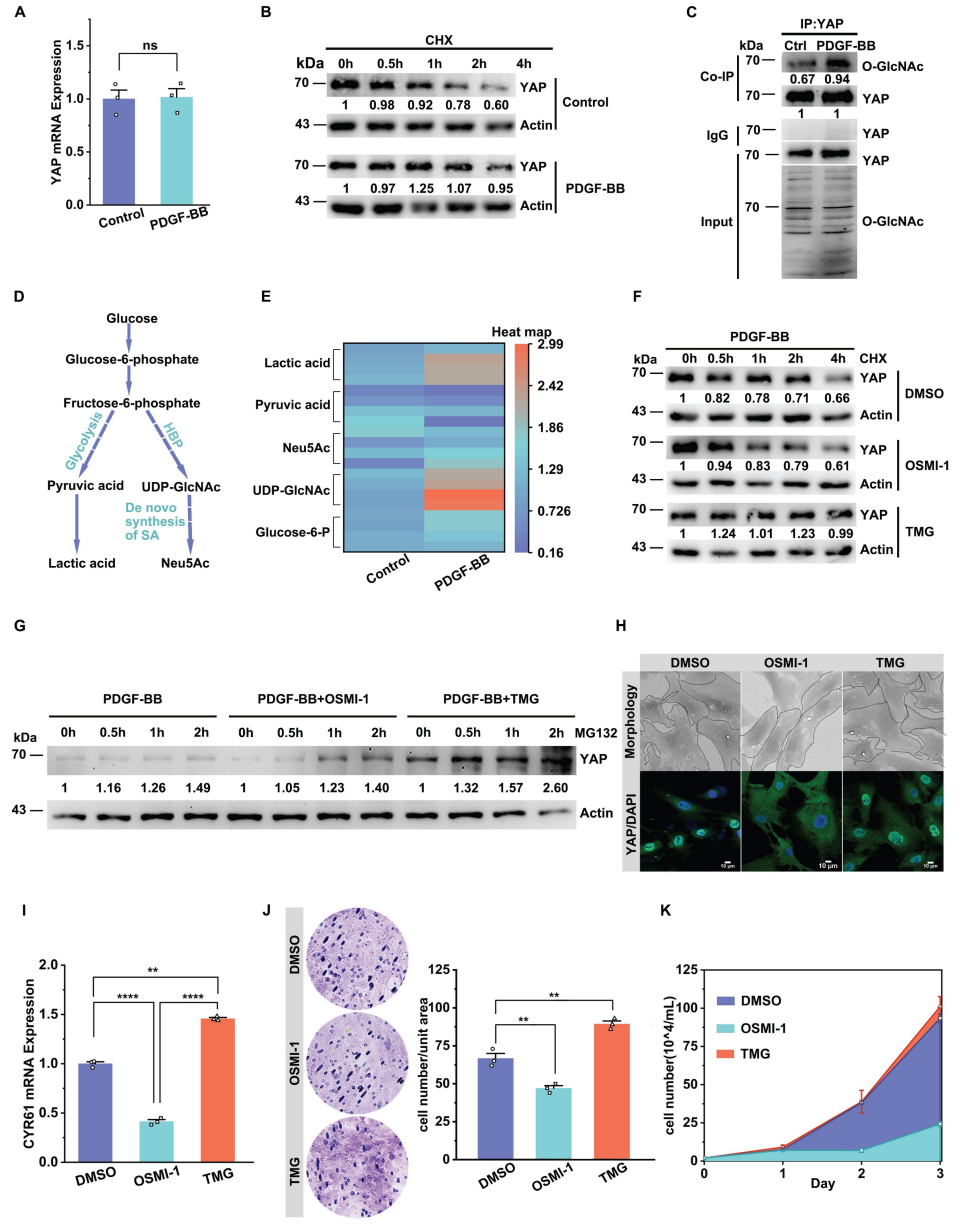
** O-GlcNAcylation enhances YAP stability and nuclear translocation to promote YAP-driven VSMC proliferation/migration.** A) YAP mRNA expression in PMSMCs with or without PDGF-BB (n = 3). Data shown as mean ± SEM, ns, no significance (one-way ANOVA with Tukey's posttest). B) Immunoblotting and quantitative analysis (relative to Actin) of YAP in PMSMCs treated with cycloheximide (20 μM) and PDGF-BB for indicated times. C) Immunoblot analysis of O-GlcNAcylated YAP in PMASMCs treated with PDGF-BB using an O-GlcNAc-specific antibody (n = 3). D) Schematic representation of the glucose metabolic flow pathway. E) LC/MS analysis heat map of various metabolites in the intracellular glucose metabolic pathway after PDGF-BB treatment (n = 4). (F-K) PMSMCs treated with PDGF-BB (10 ng/mL) and DMSO (solvent), OSMI-1 (15 μM), or TMG (10 μM) for 24 h. F) Immunoblot analysis of YAP in cells treated with cycloheximide (20 μM) for indicated time. G) Immunoblot analysis of YAP in cells treated with MG132 (10 μM) for indicated time. H) SEM images of cell morphology and immunofluorescence images of YAP (green) and DAPI (blue) (n = 3). Scale bars, 50 μm (morphology) and 10 μm (immunofluorescence). I) mRNA expression of the YAP downstream target gene representative of CYR61 (n = 3). **p < 0.01, ****p < 0.001 (one-way ANOVA with Tukey's posttest). J) Transwell Migration Assay image and the quantitative number of migrating cells indicated the migration ability of the cells (n = 3), and K) the cell counts indicated the proliferation of the cells (n = 3). **p < 0.01 (one-way ANOVA with Tukey's posttest).

First, we examined the changes in YAP gene transcription and protein translation during VSMC proliferation and migration and found no significant change in the transcript levels of YAP in both in vitro and in vivo experiments (Figure 2A, Figure S1K). However, the YAP protein level showed an increasing trend (Figure 1C & D). Therefore, we first considered whether changes in protein stability were the main reason for the increase YAP in the model group. We also examined the protein stability using a CHX experiment. The results demonstrated a significant increase in the stability of YAP after stimulation with PDGF-BB (Figure 2B).

Research has shown that the phosphorylation level of YAP leads to its ubiquitination degradation 29,54, and there is a competitive relationship between phosphorylation and O-GlcNAc glycosylation 14,20,44. Hence, we explored whether O-GlcNAcylation was one of the factors affecting YAP stability. Our results demonstrated that the O-GlcNAc level of the YAP protein was significantly increased in an in vitro model of VSMCs treated with PDGF-BB (Figure 2C). Also, the YingOYang Server predicted multiple O-GlcNAcylation sites in the YAP protein structure (Figure S2A). In addition, we detected an increase in the intracellular content of UDP-GlcNAc, an active substrate of O-GlcNAc, in the VSMC proliferation model (Figure 2D & E). We then assayed the key rate-limiting enzymes regulating the pathway and found a significant increase in the expression of GLUT1 and the O-GlcNAc transferase OGT (Figure S2B-E). OSMI-1, an inhibitor of OGT, was used to reduce protein glycosylation, and a significant decrease in YAP protein content was observed, accompanied by a relative increase in protein phosphorylation levels. The opposite results were observed after using TMG, an inhibitor of OGA (Figure S2F & G). CHX experiments showed that YAP protein decreased to 61.0% in the presence of OSMI-1, whereas in the presence of TMG, the YAP protein level remained at 99.0% after 4 h (Figure 2F). MG132 experiments showed that after 2 hours of MG132 treatment, the YAP protein levels exhibited a relative increase in both the OSMI-1 and TMG group. Notably, this increase was more pronounced in the TMG group (Figure 2G). However, OSMI-1 does not affect the mRNA levels of YAP, and TMG reduced YAP mRNA expression (Figure S2H). The above results demonstrated that O-GlcNAc modification of YAP promotes the stability of YAP protein by competitively reducing the phosphorylation level of the protein.

Next, we further investigated the relationship between O-GlcNAcylation of YAP and its nuclear translocation and VSMC proliferation and migration. We found that YAP entry into the nucleus was inhibited and accompanied by a decrease in the downstream target gene CYR61 expression after OSMI-1 intervention, and the cell morphology reverted to a shuttle shape. In contrast, the opposite occurred in the TMG-intervened group (Figure 2H & I). CCK8 (Figure S2I) and cell counting assays (Figure 2K) showed that OSMI-1 attenuated VSMC proliferation. The wound healing (Figure S2J) and Transwell Migration assays also showed that OSMI-1 attenuated the migration of VSMCs (Figure 2J). In contrast, increased VSMC proliferation and migration were observed in the presence of TMG. The above results suggest that the increased O-GlcNAcylation level of YAP in synthetic VSMCs can promote YAP protein stability, further improve its nuclear entry to activate the transcription of downstream target genes, and ultimately promote cell proliferation and migration.

### O-GlcNAcylation increases YAP binding to STAT3 to promote YAP nuclear translocation

**Figure 3:**
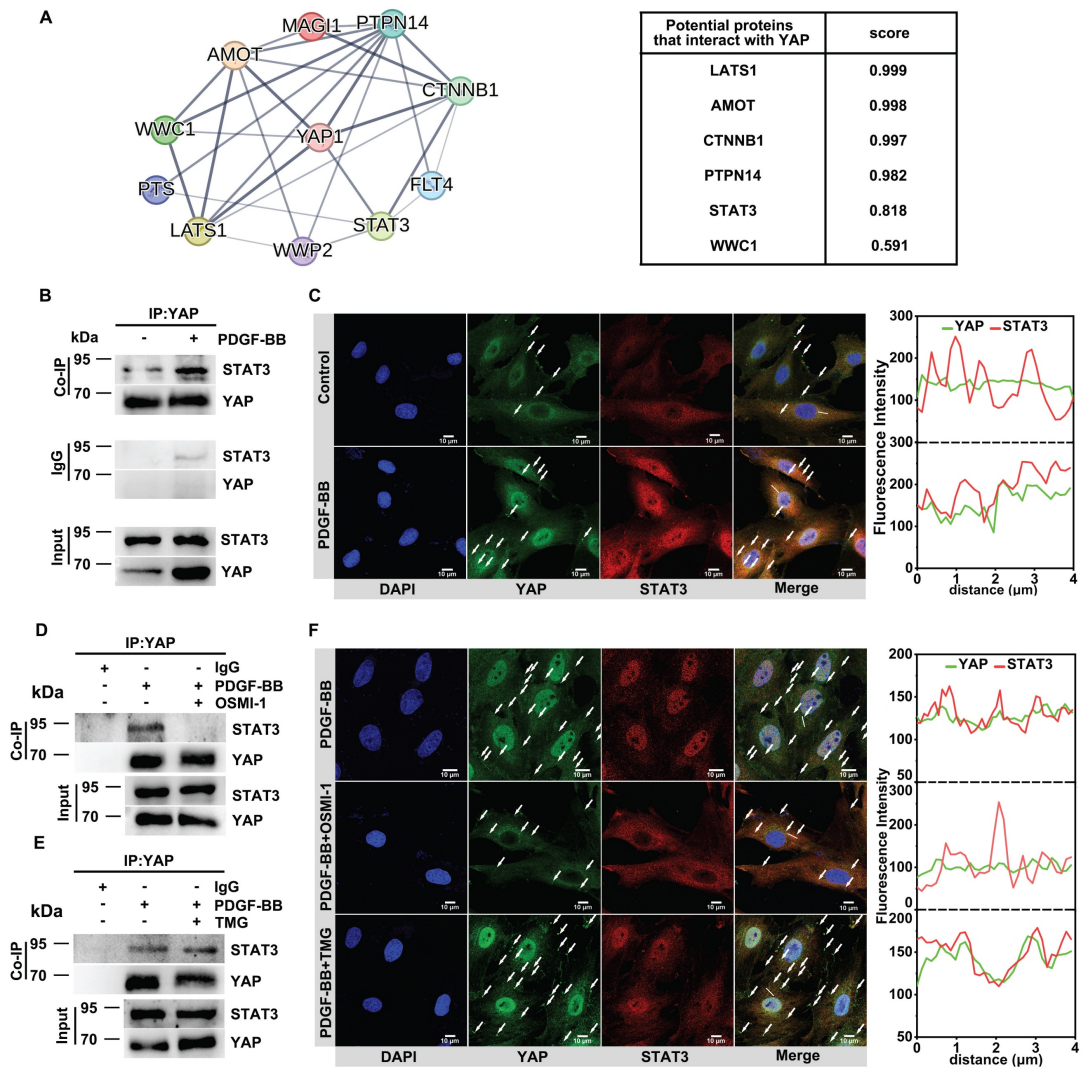
** Total O-GlcNAcylation increases YAP binding to STAT3 to promote YAP nuclear translocation.** A) YAP-interacting proteins and interaction scores predicted by the STRING server (Version: 12.0). B) Immunoblot analysis of the interaction between endogenous YAP and STAT3 in PMASMCs treated with PDGF-BB (n = 3). C) Confocal imaging of YAP (green) and STAT3 (red) co-localization in PMASMCs treated with PDGF-BB (n = 3). Scale bars, 10 µm. The arrow points to the suspected protein aggregates. The linear co-localization plots of YAP and STAT3 outlined by horizontal lines in the merged images are shown on the right. D) Immunoblot analysis of the interaction between endogenous YAP and STAT3 in PMASMCs treated with OSMI-1 (n = 3). E) Immunoblot analysis of the interaction between endogenous YAP and STAT3 in PMASMCs treated with TMG (n = 3). F) Confocal imaging of YAP (green) and STAT3 (red) co-localization in PMASMCs treated with PDGF-BB, PDGF-BB +OSMI-1 (10 μM), PDGF-BB +TMG (15 μM) (n = 3). Scale bars, 10 µm. The arrow points to the suspected protein aggregates. The linear co-localization plots of YAP and STAT3 outlined by horizontal lines in the merged images are shown on the right.

We attempted to identify how O-GlcNAcylation affects YAP nuclear translocation during VSMC proliferation and migration. PPI interaction analysis showed a potential binding between YAP and the nuclear protein STAT3 (Figure 3A). Co-IP illustrated that YAP and STAT3 were endogenously bound in the in vitro model (Figure 3B). Immunofluorescence experiments showed that a strong co-localization of the two in the cytoplasm (Figure 3C, Figure S3A). Subsequently, we found that when OSMI-1 reduced the glycosylation level of YAP, it further inhibited the endogenous binding of YAP and STAT3 and their co-localization in the cytoplasm, while TMG promoted their interaction (Figure 3D-F, Figure S3B). To further confirm that STAT3 regulates the nuclear translocation of YAP, we silenced STAT3 with siSTAT3 and found that the nuclear entry of YAP was significantly reduced (Figure S3C). These results suggested that O-GlcNAcylation may affect the nuclear translocation of YAP by regulating the binding of YAP to STAT3.

We mutated the O-GlcNAcylation site of YAP to further verify the effect of O-GlcNAcylation on YAP activation. Previous literature reported that the main sites of YAP undergoing O-GlcNAcylation are at Thr83, Ser109, Thr241, Ser334 17,55. We first carried out a homologous sequence comparison of the above sites (Figure S4A).

**Figure 4:**
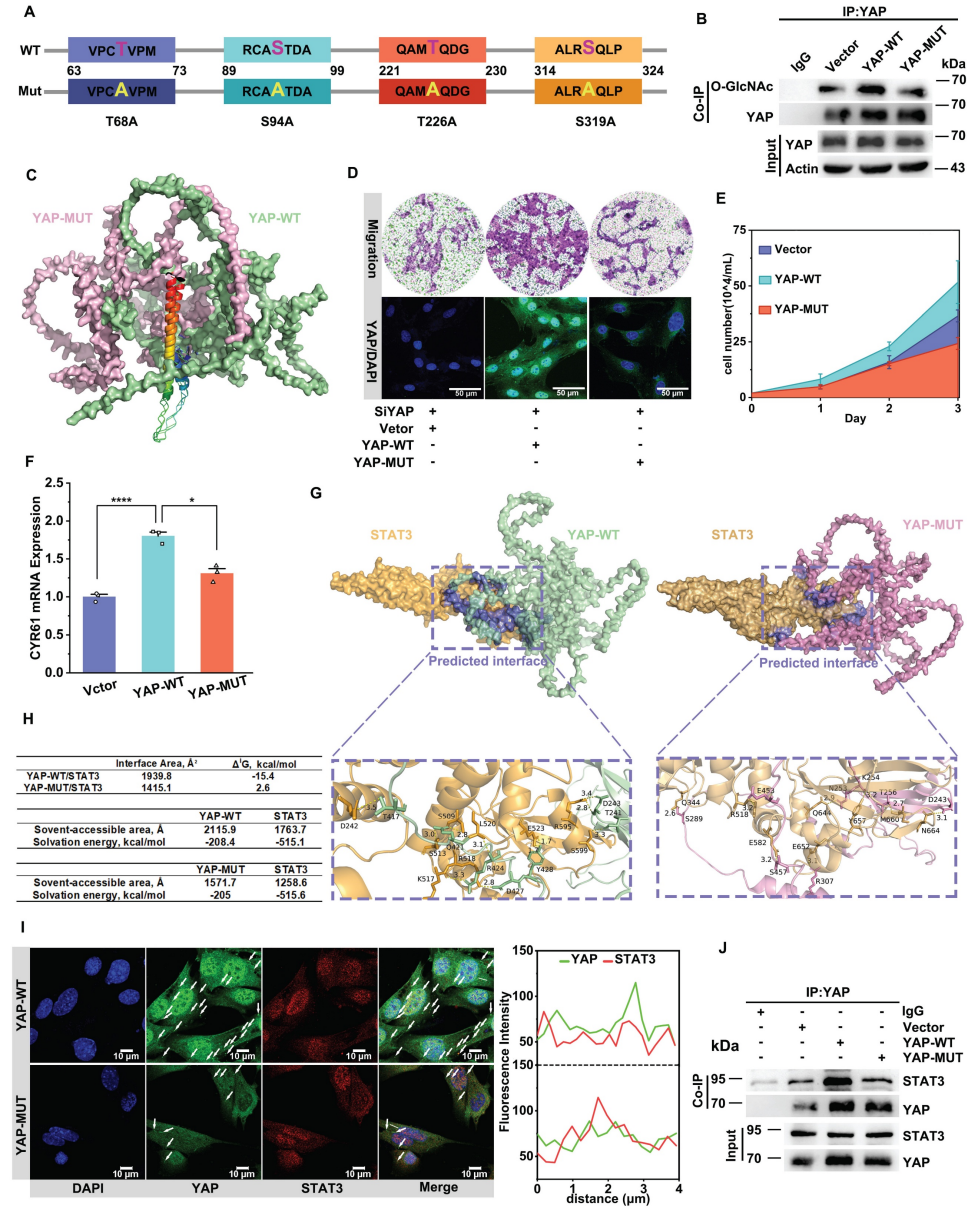
** Mutating the O-GlcNAcylation sites of YAP inhibits YAP binding to stat3 to decrease its nuclear translocation.** A) Mutation of four O-GlcNAcylated sites (S/T) on the mouse-derived YAP protein sequence to alanines. B) Immunoblot analysis of O-GlcNAcylated YAP in PMASMCs transfected with plasmids expressing Vector, wild-type (YAP-WT), or O-GlcNAcylated sites mutant (YAP-MUT) using an O-GlcNAc-specific antibody (n = 3). C) Conformational changes after full mutation of the four O-glycosylation sites Thr81, Ser109, Thr243, and Ser334 of YAP to alanines compared with YAP-WT. The colored markers are the parts of the two that are similar in conformation. (D-F, H and I) PMASMCs were transfected with plasmids expressing Vector, YAP-WT or YAP-MUT. D) Transwell Migration Assay image and immunofluorescence images of YAP (green) and DAPI (blue) of the cells (n = 3). Scale bars, 100 μm (Transwell) and 50 μm (immunofluorescence). E) Cell counts indicated the proliferation of the cells (n = 3). F) mRNA expression of YAP downstream target gene representative of CYR61 (n = 3). *p < 0.05, ***p < 0.005 (one-way ANOVA with Tukey's posttest). G) Prediction of YAP-WT or YAP-MUT protein interactions with STAT3 proteins using CoDockPP Server. The amino acid site of the binding part has been labeled. H) Docking analysis of YAP-WT or YAP-MUT proteins with STAT3 using PDBePISA Server. (I) Confocal imaging of YAP (green) and STAT3 (red) co-localization in cells (n = 3). Scale bars, 10 µm. The arrow points to the suspected protein aggregates. The linear colocalization plots of YAP and STAT3 outlined by horizontal lines in the merged images are shown on the right. J) Immunoblot analysis of the interaction between endogenous YAP and STAT3 in cells (n = 3).

Sequence alignment revealed conservation of these four loci between human and mouse YAP, so we directly mutated the four loci in the mouse sequence (Figure 4A). The O-GlcNAcylation level of YAP was significantly reduced after the loci mutation (Figure 4B, see Table S1 for the sequencing results). Then, we predicted the conformation of YAP before and after the mutation of the O-GlcNAcylation site using AlphaFold 3. The results showed that O-GlcNAc glycosylation caused a significant degree of conformational flipping of YAP, with only a small portion of the conformation remaining (Figure 4C). After mutating the O-GlcNAc sites of YAP, the results showed that the protein level of YAP decreased (Figure 4B) and the nuclear translocation of YAP was reduced (Figure 4D), accompanied by inhibition of CYR61 expression (Figure 4F). Also, the proliferation and migration of VSMCs were inhibited (Figure 4D & E, Figure S4B-D). The above results were consistent with the results of OSMI-1 action.

Finally, we verified the changes in binding between YAP and STAT3 following the mutation of the O-GlcNAc site. Through molecular docking prediction by using CodockPP server, we found that binding energy and interaction area between YAP and STAT3 were significantly reduced after mutation of O-GlcNAc site, the interaction area of YAP with STAT3 was reduced from 1,939.8 Å2 to 1,415.1 Å2, the binding energy was increased from -15.4 kcal/mol to 2.6 kcal/mol, and the number of hydrogen bonds formed was reduced from 11 to 7 (Figure 4G & H). The predicted results were verified by immunofluorescence and CO-IP experiments (Figure 4I & J, Figure S4E). Therefore, we speculate that O-GlcNAc glycosylation modification stimulates the protein binding of YAP and STAT3, thereby promoting the nuclear translocation of YAP.

The above results demonstrated that the trend of the spatiotemporal distribution of YAP and STAT3 was always consistent with the change of the O-GlcNAcylation level of YAP. When the O-GlcNAcylation level of YAP was reduced, its binding affinity to STAT3 diminished, suppressing the nuclear translocation of YAP. Consequently, YAP predominantly accumulated in the cytoplasm. In contrast, when the O-GlcNAcylation level of YAP increased, its interaction with STAT3 was strengthened, leading to enhanced nuclear translocation. Consequently, YAP was primarily localized within the nucleus. In conclusion, O-GlcNAcylation of YAP regulates YAP entry into the nucleus by promoting YAP binding to STAT3. However, the molecular mechanism by which O-GlcNAcylation regulates YAP binding to STAT3 remains to be explored.

### O-GlcNAcylation promotes YAP liquid-liquid phase separation (LLPS) to induce YAP-STAT3 condensate fusion

**Figure 5:**
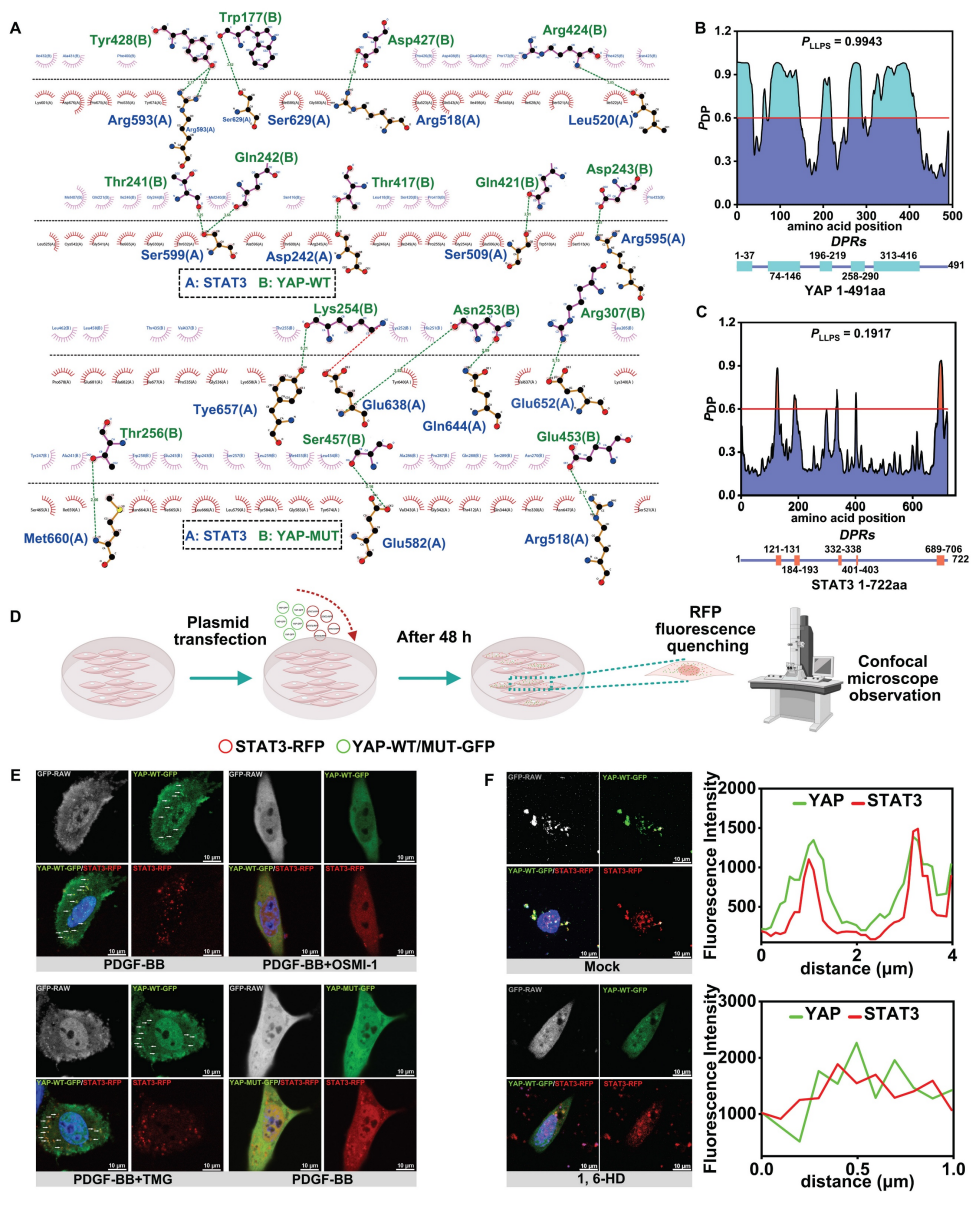
** O-GlcNAcylation promotes YAP liquid-liquid phase separation to induce YAP-STAT3 condensate fusion.** A) Demonstration of 2D planar effects of hydrogen bonds (green line) or salt bridges (red line) formed by the docking of YAP-WT proteins or YAP-MUT proteins to STAT3 proteins using LigPlot + (Version 2.2). B) Prediction of the probability of the 491 amino acids of YAP (Human) protein undergoing LLPS using FuzDrop server. The horizontal line indicates PDP≥0.6. Identification of droplet-promoting regions (DPRs) within the YAP protein structure that may undergo LLPS. C) Prediction of the probabilities of LLPS for the 722 amino acids of STAT3 (Human) protein using FuzDrop server. D) Experimental procedure for the observation of protein LLPS phenomenon by confocal microscopy. E) PMASMCs co-transfected with YAP-WT-GFP and STAT3-RFP plasmids in the first three groups. The fourth group co-transfected with YAP-MUT-GFP and STAT3-RFP plasmids and treated with PDGF-BB and various compounds for 12 h. Droplet formation of YAP and STAT3 detected using confocal microscope. Scale bars, 10 µm. F) PMASMCs were co-transfected with YAP-WT-GFP and STAT3-RFP plasmids for 48 h, then treated with PBS or 1.5% 1,6-hexanediol (1,6-HD) for 2 min and observed by confocal microscope. Scale bars, 10 µm. The linear colocalization plots of YAP and STAT3 outlined by horizontal lines in the merged images are shown on the right.

We attempted to explore the specific mechanism by which O-GlcNAc affects the binding of YAP to STAT3. The CodockPP server predicted the interaction between YAP and STAT3. The mutation of the O-GlcNAc site of YAP resulted in a decrease in the number of hydrogen bonds between YAP and STAT3, from 11 to 7 (Figure 5A, Figure 4F). immunofluorescence staining of YAP and STAT3 illustrated the formation of protein aggregates (Figure 3C & F, Figure 4I). Studies have confirmed that charge-charge-based hydrogen bonds are typical interaction forces that mediate protein LLPS 56,57. Therefore, we hypothesized that the O-GlcNAcylation may affect the interaction of YAP with STAT3 by influencing the LLPS. We first found that both YAP and STAT3 were enriched in IDRs as predicted from the FuzDrop server, and the structural domains with PDPs greater than 0.6 suggested that the two had a strong LLPS propensity (Figure 5B & C). Moreover, the three glycosylation sites of YAP, T81, S109 and S334, were in the predicted disordered structural domains. We further speculated that O-GlcNAcylation of YAP might be involved in the LLPS of YAP.

Subsequently, we co-transfected YAP-WT-GFP and STAT3-RFP plasmids with different fluorescent tags (Figure 5D). The results showed droplet formation in both YAP and STAT3 under the intervention of PDGF-BB and TMG, and the Pearson correlation coefficients suggested that the two formed droplets with a significant co-localization (Figure 5E, Figure S5A). On the contrary, YAP droplet formation was reduced or almost absent after OSMI-1 action and transfection of YAP glycosylation site mutant plasmid YAP-MUT-GFP. However, STAT3 droplet formation was not affected by the O-GlcNAcylation and only attenuated the co-localization of YAP with STAT3 (Figure 5E, Figure S5A).

Next, we performed red fluorescence quenching experiments on YAP-WT-GFP, YAP-MUT-GFP, and STAT3-RFP droplets in vitro using photobleaching. We found that the fusion time of YAP and STAT3 droplets was prolonged after mutating the sites (Figure S5B & C). To further verify the effect of LLPS on the binding of YAP and STAT3, 1, 6-hexanediol (1,6-HD), an inhibitor of LLPS, was used. The results showed that inhibition of LLPS of YAP and STAT3 significantly weakened the co-localization between them (Figure 5F, Figure S5D). The above experimental results strongly suggested that an increased level of O-GlcNAcylation of YAP promotes the LLPS of YAP and subsequently accelerates the fusion of YAP condensate with STAT3 condensate, enhancing the interaction between YAP and STAT3.

### Natural medicine small molecules can effectively delay neointima formation by targeting YAP

**Figure 6:**
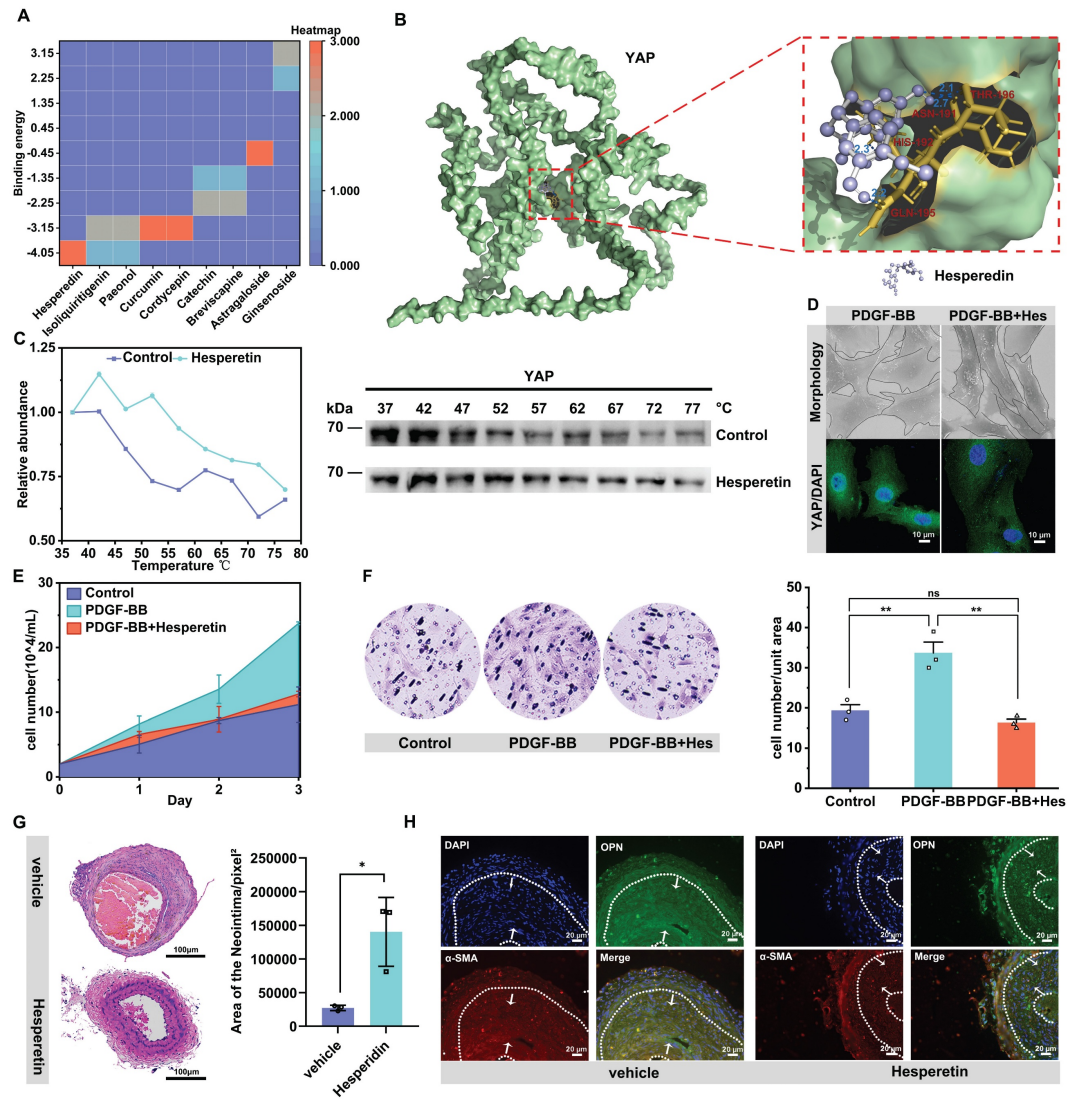
** Natural medicine small molecules can effectively delay neointima formation by targeting YAP.** A) Heat map of binding energy of 9 small molecules docking with YAP. B) Molecular docking diagram of hesperidin and YAP, with the dotted boxes of specific binding regions and amino acid sites enlarged on the right, and the dashed blue lines indicating hydrogen bonds. C) Cellular Thermal Shift Assay (CETSA) showing the stability of YAP under Control or hesperidin (100 μM) treatment at various temperatures. (D-F) PMASMCs were treated with PDGF-BB or PDGF-BB and Hesperidin. D) SEM images of cell morphology and immunofluorescence images of YAP (green) and DAPI (blue) for cells (n = 3). Scale bars, 50μm (morphology) and 10μm (immunofluorescence). E) The cell count indicated the proliferation of the cells. (F) The Transwell Migration Assay image and the quantitative number of migrating cells indicated the migration ability of the cells (n = 3). **p < 0.01, ns, no significance (one-way ANOVA with Tukey's posttest). G) HE staining of the left carotid artery of mice with corn oil (Vehicle group) or hesperidin (50 mg/kg/d) for 21 days was performed from the site of ligation to the proximal end to visualize the formation of neointima (n = 3). Scale bars, 100μm. On the right is a quantitative graph of the area of the neointima, with the unit of pixels^2^. H) Immunofluorescence detection of VSMCs phenotypic transforming factors in paraffin sections. Scale bars, 20μm.

We explored whether known natural drug small molecules, which have been reported to inhibit cell proliferation and migration, also inhibit vascular neointima formation by targeting YAP 58-62. Molecular docking of 9 small molecules with YAP was performed, and the results showed that the flavonoid small molecule hesperidin had the smallest binding energy with YAP and was one of the most potent molecules targeting YAP (Figure 6A & B). We further verified through thermal stability experiments that hesperidin could bind to YAP (Figure 6C). We thus speculated that YAP might be one of the targets of hesperidin to inhibit the proliferation and migration of VSMCs. We selected 100 μM hesperidin by CCK-8 for in vitro experiments (Figure S6A). Under the intervention of hesperidin, the nucleation of YAP was inhibited, and the VSMCs were transformed into a contractile form with a shuttle morphology (Figure 6D). Hesperidin inhibited the proliferation and migration of VSMCs (Figure 6E, F, Figure S6A, B). Also, hesperidin may regulate YAP entry into the nucleus by influencing the binding of YAP to STAT3 (Figure S6D-F). Interestingly, hesperidin promoted the transcriptional level of YAP but decreased YAP protein expression (Figure S6C, D). We considered the possibility that hesperidin affected the stability of YAP, so we examined the O-GlcNAcylation of YAP under hesperidin intervention and surprisingly found that hesperidin also had an inhibitory effect on the O-GlcNAcylation of YAP (Figure S6G), suggesting that the binding of hesperidin to YAP may affect the nuclear translocation of the protein by hindering its glycosylation modification.

**Figure 7:**
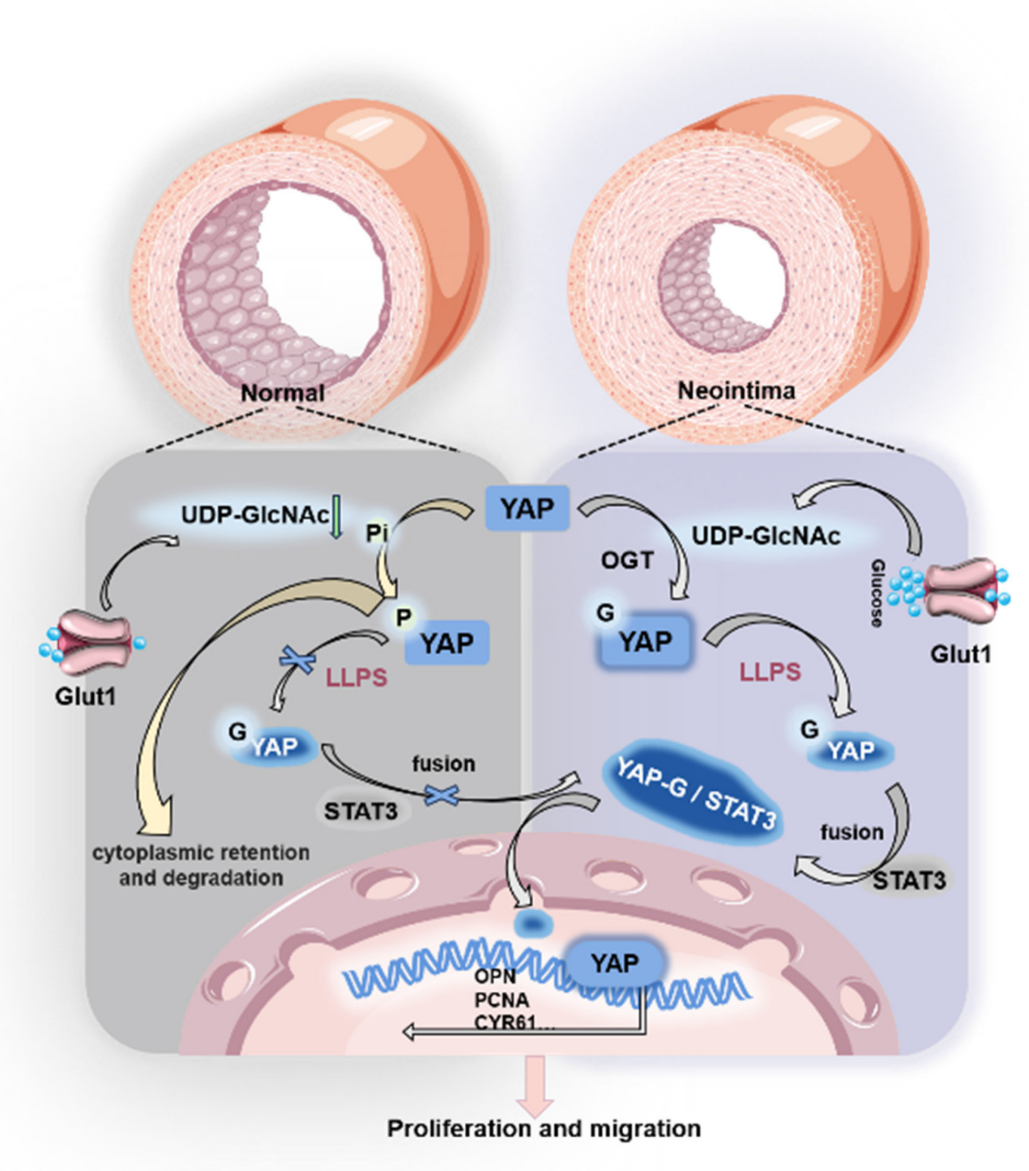
Mechanism diagram.

To verify whether hesperidin inhibits neointimal formation in vivo, we fed hesperidin (50 mg/kg/d) by gavage for 21 days to mice undergoing carotid artery ligation. As expected, the results showed that hesperidin significantly inhibited the proliferation of vascular neointima (Figure 6G). Immunofluorescence analysis of paraffin sections showed that hesperidin treatment reversed the phenotypic transformation of VSMCs (Figure 6H). Furthermore, the YAP protein level and its nuclear localization were reduced after hesperidin intervention, and the co-localization of YAP with STAT3 was also blocked (Figure S6H). The surgical operation and the dose of hesperidin throughout the process did not significantly affect the body weight of the mice (Figure S6I), and there was no significant organ damage (Figure S6J). The main mechanism of this study is illustrated in Figure 7.

## Discussion

O-GlcNAc glycosylation is an important post-translational modification (PTM) that plays a key role in regulating tumor cell proliferation and migration. O-GlcNAc glycosylation controls critical cellular processes through mechanisms such as protein stabilization, modulation of interactions, and competitive inhibition of phosphorylation 63. Our experimental results in the vascular restenosis model indicate that O-GlcNAc glycosylation affects the stability and activity of key proteins such as YAP, affecting the proliferation and migration of VSMCs. However, the mechanism by which O-GlcNAc glycosylation regulates YAP nuclear import is unclear 17,25,55. YAP lacks conventional nuclear translocation signals, but reports have confirmed that YAP can enter the nucleus with the cooperative action of STAT3 45. Our experimental results demonstrated that the O-GlcNAc glycosylation modification of YAP regulates the interaction between YAP and STAT3. Significantly elevated levels of UDP-GlcNAc, a substrate for intracellular glucose-6-phosphate and O-GlcNAc glycosylation, were detected in a restenosis model by LC/MS, resulting in enhanced O-GlcNAc glycosylation of YAP. Subsequently, using the inhibitor OSMI-1 of OGT (the key enzyme of O-GlcNAc glycosylation) or mutating the glycosylation site of YAP could reduce the interaction between YAP and STAT3, and at the same time, YAP remained in the cytoplasm. On the contrary, after using TMG, an inhibitor of O-GlcNAc hydrolase OGA, the O-GlcNAc glycosylation of YAP increased, and enhanced co-localization of YAP and STAT3 was observed. Both YAP and STAT3 co-translocated into the nucleus to activate downstream pro-proliferation and migration genes. Thus, our results clarified the regulatory mechanism of O-GlcNAc modification in the YAP nuclear translocation process and its impact on diseases.

Liquid-liquid phase separation is prevalent in eukaryotic cells. It has recently been suggested to be critical for regulating various biological processes, including transcription, translation, expression, post-transcriptional and post-translational modifications, cell signal transduction, and protein-protein interactions, by compartmentalizing proteins or nucleic acids into droplet-like condensates 33,34,37,64. Post-translational modification (PTM) of proteins has emerged as a key link in the regulation of LLPS under physiological or pathophysiological conditions 36,39,40. Two research groups found that mTORC1-mediated phosphorylation promotes LLPS in PGLs, preventing autophagic degradation 65. Another group also found that SUMOylation was involved in the LLPS of promyelocytic leukemia protein nucleosomes (PML-NBs) 66. Our present study found that O-GlcNAc-mediated LLPS is essential in regulating YAP. The O-GlcNAcylation of YAP promoted the accumulation of YAP protein aggregates and increased the compartmentalization between YAP and STAT3, impacting the proliferative effect of YAP. Our results also revealed that decreasing the O-GlcNAcylation level of YAP inhibited YAP from undergoing LLPS and prevented it from binding to STAT3 and entering the nucleus. On the contrary, increasing the O-GlcNAcylation of YAP resulted in the formation of YAP protein condensates and promotes the binding of YAP to STAT3 for nuclear translocation. Then, after the treatment with the LLPS inhibitor 1,6-hexadiol, the protein condensate of YAP gradually disappeared, and with the reduced binding to STAT3, the nuclear translocation was inhibited. These results indicated that LLPS plays a vital role in the binding of YAP and STAT3, which is influenced by O-GlcNAcylation.

YAP plays a crucial role in cell signaling and gene regulation through LLPS in the cytoplasm and the nucleus. In the cytoplasm, YAP forms condensates via LLPS, promoting its interaction with other signaling molecules, thereby influencing the activation of signaling pathways. For example, YAP condensates in the cytoplasm can interact with key components of the Hippo pathway, such as LATS1/2 and NLK, to regulate the phosphorylation and nuclear translocation of YAP 32. In the nucleus, the condensates formed by YAP through LLPS can facilitate its interaction with transcription factors like TEADs, thus enhancing the transcriptional regulation of its target genes, such as CTGF and CYR61. These condensates can enrich transcription factors and co-activators, improving transcriptional efficiency 28,67,68. Research on the mechanism of YAP nuclear translocation in the cytoplasmic region where LLPS occurs is still lacking.

In our research, we uncovered the regulatory mechanism by which O-GlcNAcylation modification influences the nuclear entry of YAP. The O-GlcNAcylation facilitates the LLPS process of YAP, enhances the binding of YAP to the STAT3 protein, and subsequently, with the assistance of STAT3, YAP enters the nucleus. The LLPS of YAP in the cytoplasm and the nucleus not only contributes to its functions in signal transduction and transcriptional regulation but also improves the efficiency of its interaction with other molecules by forming condensates, thereby playing an essential role in cell fate determination and tissue development. These findings provide new insights into understanding the complex mechanisms of YAP in cell signaling and gene regulation and offer potential targets for treating related diseases.

Emerging research shows that natural drug small molecules, including hesperidin, quercetin and other flavonoids, can reduce the proliferation and migration of VSMCs 69. However, its specific target and pharmacodynamic mechanism have not yet been fully elucidated. In our study, hesperidin showed a high affinity to YAP in both *in vitro* experiments and virtual molecular docking, suggesting that YAP is a potential target of hesperidin. Consistent with the *in vitro* observations, *in vivo* experiments verified that hesperidin significantly inhibited neointima formation and slowed down carotid artery stenosis in mice. Hesperidin could regulate YAP nuclear translocation by inhibiting the binding of YAP to STAT3, but whether the specific mechanism is related to O-GlcNAc-dependent LLPS separation requires further study and will be explored in our future studies. Nevertheless, our current findings provide new insights into the potential clinical application of hesperidin in alleviating the proliferation and migration of VSMCs.

In conclusion, we identified a potential mechanism by which YAP O-GlcNAcylation promotes VSMC proliferation and migration. The specific mechanism is that O-GlcNAc of YAP fosters the formation of YAP condensates, strengthens the interaction between YAP and the nucleation helper protein STAT3, stimulates the proliferation and migration of VSMCs by promoting YAP entry into the nucleus, and thus accelerates the formation of neointima. The small-molecule drug hesperidin can target YAP to achieve pharmacological effect of inhibiting neointima formation, which provides potential clinical application for more natural drugs to inhibit neointima formation of blood vessels with small molecules, and provides a new idea for the treatment of carotid artery stenosis from the perspective of regulating LLPS.

## Supplementary Material

Supplementary figures and table.
