# Supplementary material for: O-GlcNAcylation-dependent liquid-liquid phase separation regulates the nuclear translocation of YAP to exacerbate vascular neointimal hyperplasia
Source: Theranostics. 2025 Jul 11;15(16):7956–72. doi: 10.7150/thno.113303 (PMC12374541; doi:10.7150/thno.113303)
Supplement: Supplementary file 1 — Supplementary figures and table. [file thnov15p7956s1.pdf]

Supplementary Information

O-GlcNAcylation-dependent liquid-liquid phase separation regulates nuclear translocation of YAP to exacerbate vascular neointimal hyperplasia

Ping Weng[1,2*], Yilin Wen[1,2*], Zhiyi Yuan[1,2], Limei Ma[1,2], Liming Yang[1,2], Chengju Li[1,2], Wanping Zhang[1,2], Chao Yu[1,2]

1. College of Pharmacy, Chongqing Medical University, Chongqing, 400016, China.

2. Chongqing Key Laboratory for Pharmaceutical Metabolism Research, Chongqing,400016, China.

* Ping Weng, and Yilin Wen contributed equally to this work and share first authorship.

#Corresponding Author: Chao Yu, Ph.D. and M.D. (yuchao@cqmu.edu.cn (C. Yu))

This supplementary information contains:

- Supplementary Table
- Supplementary Figures S1 to S6.

Supplementary Table

Table S1. Base sequences of wild-type and mutant plasmids of the house mouse YAP

**Name**	**Base sequences**
**YAP-WT**	**GCTAGCgccaccATGGAGCCCGCGCAACAGCCGCCGCCCCAGCC GGCCCCGCAAGGCCCCGCGCCGCCGTCCGTGTCTCCGGCCGG GACCCCCGCGGCCCCGCCCGCACCCCCGGCCGGCCACCAGGT CGTGCACGTCCGCGGGGACTCGGAGACCGACTTGGAGGCGCT CTTCAATGCCGTCATGAACCCCAAGACGGCCAACGTGCCTCA GACCGTGCCCATGCGGCTTCGCAAGCTGCCCGACTCCTTCTT CAAGCCGCCTGAGCCCAAGTCCCACTCGCGACAGGCCAGTAC TGATGCAGGTACTGCGGGAGCTCTGACTCCACAGCATGTTCG AGCTCACTCCTCTCCAGCCTCCCTGCAGCTGGGTGCCGTTTCT CCTGGGACACTCACAGCCAGTGGCGTTGTCTCTGGCCCTGCC GCTGCCCCTGCAGCTCAGCATCTCCGGCAGTCCTCCTTTGAG ATCCCTGATGATGTACCACTGCCAGCAGGCTGGGAGATGGCCA AGACATCTTCTGGTCAAAGATACTTCTTAAATCACAACGATCA GACAACAACATGGCAGGACCCCCGGAAGGCCATGCTTTCGCA ACTGAACGTTCCTGCGCCTGCCAGCCCAGCGGTGCCCCAGAC GCTGATGAATTCTGCCTCAGGACCTCTTCCTGATGGATGGGAG CAAGCCATGACTCAGGATGGAGAAGTTTACTACATAAACCATA AGAACAAGACCACATCCTGGCTGGACCCAAGGCTGGACCCTC**

	**GTTTTGCCATGAACCAGAGGATCACTCAGAGTGCTCCAGTGAA GCAGCCCCCACCCTTGGCTCCCCAGAGCCCACAGGGAGGCGT CCTGGGTGGAGGCAGTTCCAACCAGCAGCAGCAAATACAGCT GCAGCAGTTACAGATGGAGAAGGAGAGACTGCGGTTGAAACA ACAGGAATTATTTCGGCAGGAATTAGCTCTGCGCAGCCAGTTG CCTACACTGGAGCAGGATGGAGGGACTCCGAATGCAGTGTCT TCTCCTGGGATGTCTCAGGAATTGAGAACAATGACAACCAATA GTTCCGATCCCTTTCTTAACAGTGGCACCTATCACTCTCGAGA TGAGAGCACAGACAGCGGCCTCAGCATGAGCAGCTACAGCAT CCCTCGGACCCCAGACGACTTCCTCAACAGTGTGGATGAAAT GGATACAGGAGACACCATCAGCCAAAGCACCCTGCCGTCACA GCAGAGCCGCTTCCCCGACTACCTGGAAGCCCTCCCTGGGAC AAATGTGGACCTTGGCACACTGGAAGGAGATGCAATGAACATA GAAGGGGAGGAGCTGATGCCCAGTCTGCAGGAAGCGCTGAGT TCCGAAATCTTGGACGTGGAGTCTGTGTTGGCTGCCACCAAG CTAGATAAAGAAAGCTTTCTCACGTGGTTAGACTACAAAGACG ATGACGACAAGTAGGGATCC**
**YAP-MUT**	**GCTAGCgccaccATGGAGCCCGCGCAACAGCCGCCGCCCCAGCC GGCCCCGCAAGGCCCCGCGCCGCCGTCCGTGTCTCCGGCCGG GACCCCCGCGGCCCCGCCCGCACCCCCGGCCGGCCACCAGGT CGTGCACGTCCGCGGGGACTCGGAGACCGACTTGGAGGCGCT CTTCAATGCCGTCATGAACCCCAAGACGGCCAACGTGCCTCA**

	**GGCCGTGCCCATGCGGCTTCGCAAGCTGCCCGACTCCTTCTT<br>CAAGCCGCCTGAGCCCAAGTCCCACTCGCGACAGGCCgccACT<br>GATGCAGGTACTGCGGGAGCTCTGACTCCACAGCATGTTCGA<br>GCTCACTCCTCTCCAGCCTCCCTGCAGCTGGGTGCCGTTTCTC<br>CTGGGACACTCACAGCCAGTGGCGTTGTCTCTGGCCCTGCCG<br>CTGCCCCTGCAGCTCAGCATCTCCGGCAGTCCTCCTTTGAGAT<br>CCCTGATGATGTACCACTGCCAGCAGGCTGGGAGATGGCCAA<br>GACATCTTCTGGTCAAAGATACTTCTTAAATCACAACGATCAG<br>ACAACAACATGGCAGGACCCCCGGAAGGCCATGCTTTCGCAA<br>CTGAACGTTCCTGCGCCTGCCAGCCCAGCGGTGCCCCAGACG<br>CTGATGAATTCTGCCTCAGGACCTCTTCCTGATGGATGGGAGC<br>AAGCCATGgccCAGGATGGAGAAGTTTACTACATAAACCATAAG<br>AACAAGACCACATCCTGGCTGGACCCAAGGCTGGACCCTCGT<br>TTTGCCATGAACCAGAGGATCACTCAGAGTGCTCCAGTGAAG<br>CAGCCCCCACCCTTGGCTCCCCAGAGCCCACAGGGAGGCGTC<br>CTGGGTGGAGGCAGTTCCAACCAGCAGCAGCAAATACAGCTG<br>CAGCAGTTACAGATGGAGAAGGAGAGACTGCGGTTGAAACAA<br>CAGGAATTATTTCGGCAGGAATTAGCTCTGCGCgctCAGTTGCC<br>TACACTGGAGCAGGATGGAGGGACTCCGAATGCAGTGTCTTC<br>TCCTGGGATGTCTCAGGAATTGAGAACAATGACAACCAATAGT<br>TCCGATCCCTTTCTTAACAGTGGCACCTATCACTCTCGAGATG<br>AGAGCACAGACAGCGGCCTCAGCATGAGCAGCTACAGCATCC**

	**CTCGGACCCCAGACGACTTCCTCAACAGTGTGGATGAAATGG<br>ATACAGGAGACACCATCAGCCAAAGCACCCTGCCGTCACAGC<br>AGAGCCGCTTCCCCGACTACCTGGAAGCCCTCCCTGGGACAA<br>ATGTGGACCTTGGCACACTGGAAGGAGATGCAATGAACATAGA<br>AGGGGAGGAGCTGATGCCCAGTCTGCAGGAAGCGCTGAGTTC<br>CGAAATCTTGGACGTGGAGTCTGTGTTGGCTGCCACCAAGCT<br>AGATAAAGAAAGCTTTCTCACGTGGTTAGACTACAAAGACGAT<br>GACGACAAGTAGGGATCC**

Supplementary Figures

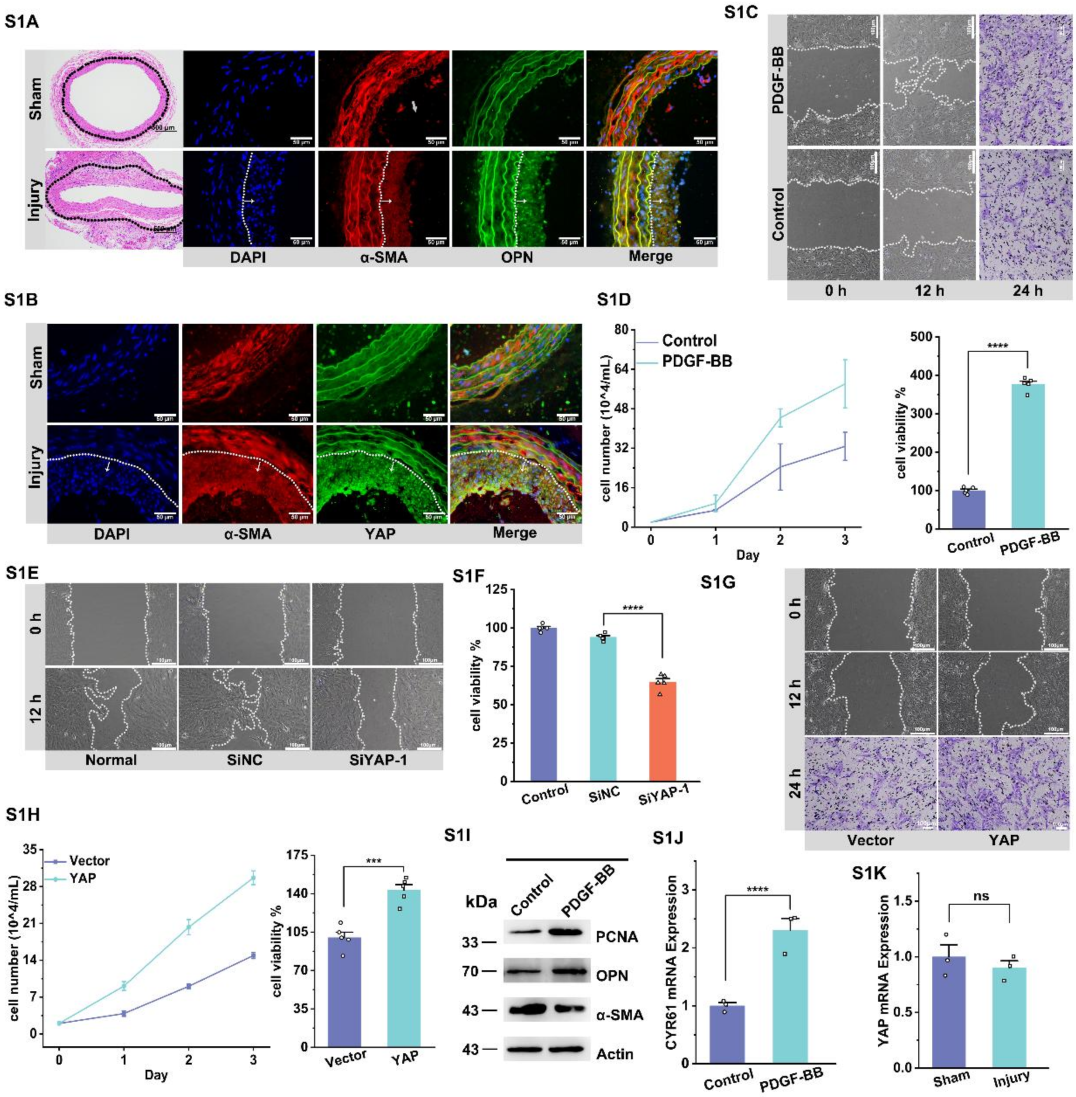

Figure S1. Validation of experimental phenotypes *in vitro* and *in vivo*. (A) HE detection of vascular neointima formation in sham-operated (Sham group) and operated (Injury group) rats 14 d after balloon injury. Scale bar, 500 μm. Phenotypic transformation of vascular smooth muscle cells (VSMC) in Sham and Injury groups after 14 d of balloon injury in rats by immunofluorescence on frozen sections. α-SMA was the stationary marker and OPN was the synthetic marker. scale bar, 50 μm. (B) Immunofluorescence detection of YAP expression in VSMCs in Sham and Injury groups of rats 14 d after balloon injury in frozen sections. scale bar, 50 μm. (C-D)

Primary mouse aortic smooth muscle cells (PMASMCs) were treated with PDGF-BB (10 ng/mL) for 24h. (C) Wound healing assay measured the change in distance of cells cultured in 5% serum medium for 12 h. Scale bar, 100 μm. Transwell migration assay measured the number of cells that migrated to the lower compartment within 24 h. (D) Cells were counted (n = 3). Changes in OD values of cells at 450 nm wavelength were measured by CCK8 assay (n = 5). **** = p < 0.001 (unpaired, two-tailed student's test). (E) Wound healing assay measured the change in distance of cells after SiYAP silencing PMASMCs indicated the migration ability of the cells and (F) the CCK8 assay indicated the proliferation of the cells (n = 5). **** = p < 0.001 (unpaired, two-tailed student's test). (G) Wound healing and Transwell migration assays showed the migration rate of plasmid-mediated YAP overexpression in PMASMCs. (H) Cell counts and CCK8 assays showed the growth rate of YAP overexpression in PMASMCs for 0, 1, 2, and 3 days (n = 3 and n = 5, respectively). *** = p < 0.005 (unpaired, two-tailed student's test).(I) Western blot was used to detect the protein levels of PCNA, α-SMA, OPN, PCNA is a proliferative marker, α-SMA is a quiescent marker, and OPN is a synthetic marker. (J) RT-PCR was performed to detect mRNA levels of the target gene CYR61 downstream of YAP (n = 3). **** = p < 0.001 (one-way ANOVA with Tukey's posttest). (K) Indicates changes in YAP mRNA levels in Sham group and Injury group after 21 d of common carotid artery ligation in mice (n = 3). ns=no significance (one-way ANOVA with Tukey's posttest).

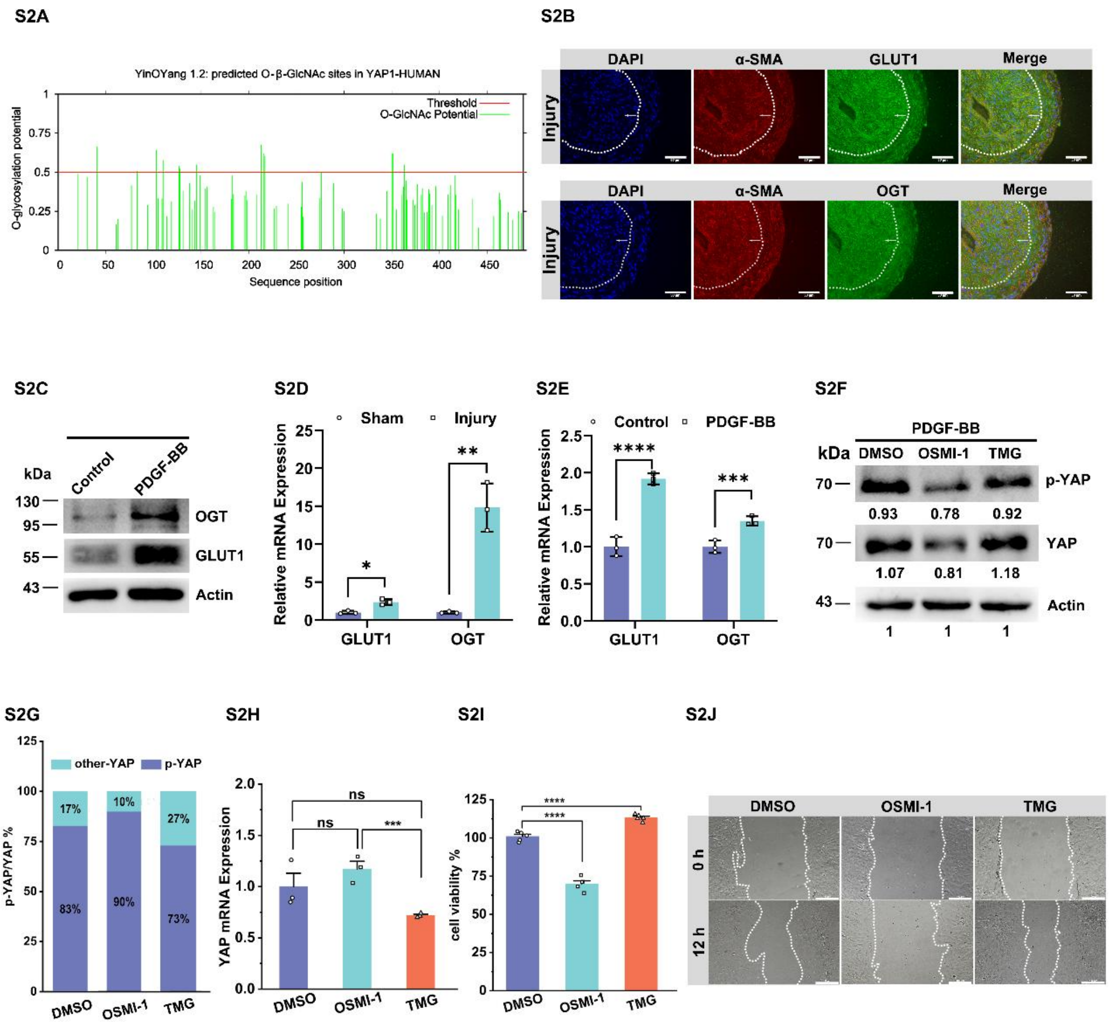

Figure S2. (A) YingOYang server predicted the O-GlcNAc sites where human-derived YAP may occur. (B) Immunofluorescence detection of the expression levels of glucose transporter protein 1 (GLUT1) and O-GlcNAc transferase (OGT) in the neointima of mouse carotid arteries after ligation for 21 d. Inside the dotted line is the neointima, and outside the dotted line is the intima media of the original vessel. (C) Protein levels of OGT and GLUT1 were detected by Western blot in PASMCs were treated with PDGF-BB (10 ng/mL) for 24 h. (D) Indicates changes in GLUT1 and OGT mRNA levels in Sham group and Injury group after 21 d of common carotid artery ligation in mice (n = 3). * =p < 0.05, ** =p < 0.01. (E) Indicates changes in GLUT1 and OGT mRNA levels

in cells treated with PDGF-BB after 24 h (n = 3). *** =p < 0.005, **** =p < 0.001. (one-way ANOVA with Tukey's posttest).(F-J) PASMCs were treated with PDGF-BB (10 ng/mL) and DMSO, OSMI-1 (15 μM), or TMG (10 μM), respectively, for 24 h.(F) Protein levels of YAP and p-YAP. (G) Quantified phosphorylation levels of YAP (Ser127) normalized to total YAP protein in (F). (H) mRNA level of YAP (n = 3). Data are mean±SEM, ns=no significance, *** = p < 0.005 (one-way ANOVA with Tukey's posttest). (I) the CCK8 indicated the proliferation of the cells (n = 5) and (J) Wound healing indicated the migration ability of the cells (n = 3). **** = p < 0.001 (one-way ANOVA with Tukey's posttest).

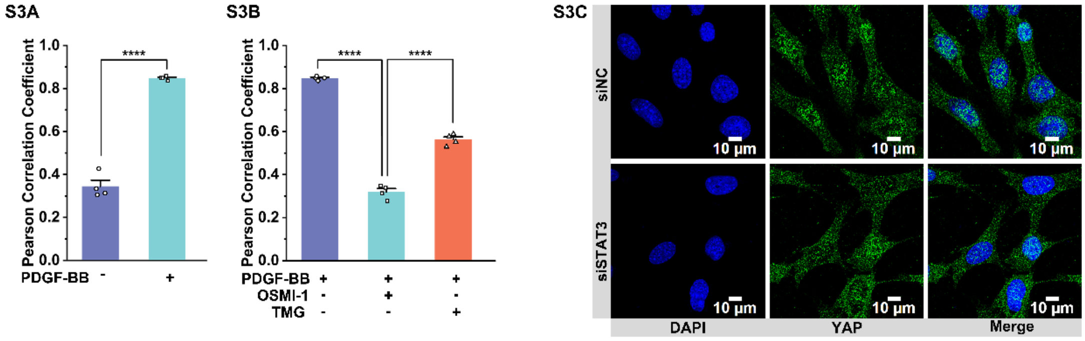

Figure S3. (A) Pearson correlation coefficient between YAP and STAT3 in cytoplasm in Figure 3C (n = 4). **** = p < 0.001 (unpaired, two-tailed student's test). (B) Pearson correlation coefficient between YAP and STAT3 in cytoplasm in Figure 3F (n = 4). **** = p < 0.001 (unpaired, two-tailed student's test). (C) Confocal imaging was performed to observe the nuclear translocation of YAP (green) in PMASMCs treated with siSTAT3 (n = 3). Scale bar, 10 μm.

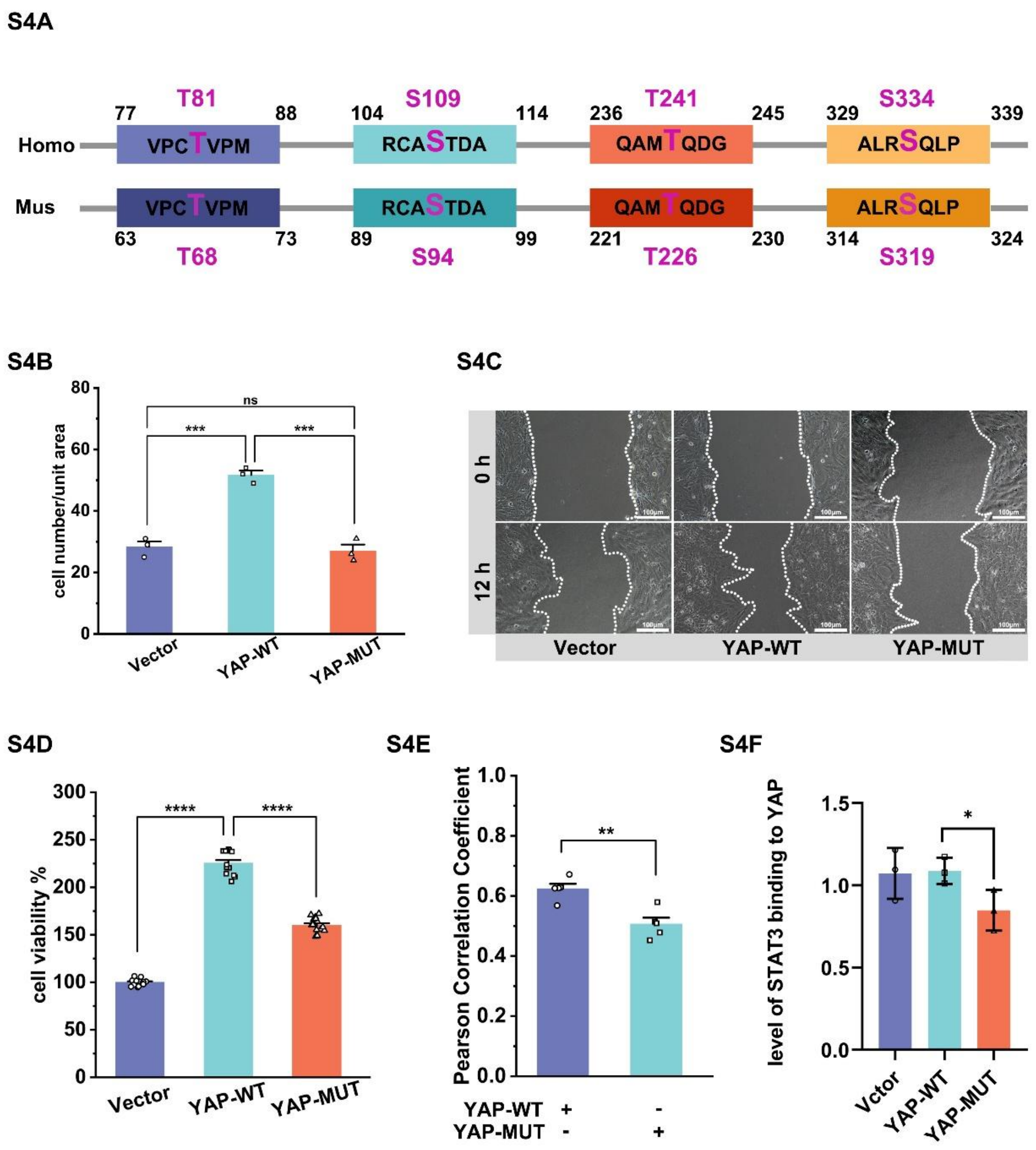

Figure S4. (A) Comparison of the Amino acid sequences showing the four O-GlcNAcylation sites of human and mouse YAP. Sites where significant O-GlcNAcylation occurs are marked with purple. (B) cell number of Transwell assay in Figure 4D (n = 3). ns=no significance, *** = $p < 0.005$ (unpaired, two-tailed student's test). (C) Wound healing assay showed the migration rate of cells (n = 3) and (D) CCK8 assay indicated the growth rate of PMASMCs (n = 5), which transfected with empty (Vector), YAP-WT, and YAP-MUT plasmids. Scale bar,100 μm. **** = $p < 0.001$

(unpaired, two-tailed student's test). (E) Pearson correlation coefficient between YAP and STAT3 in cytoplasm in Figure 4I (n = 4). ** = p < 0.01 (unpaired, two-tailed student's test). (F) the protein quantification graph of STAT3 bound to YAP in Figure 4J, n = 3, * indicates P < 0.05.

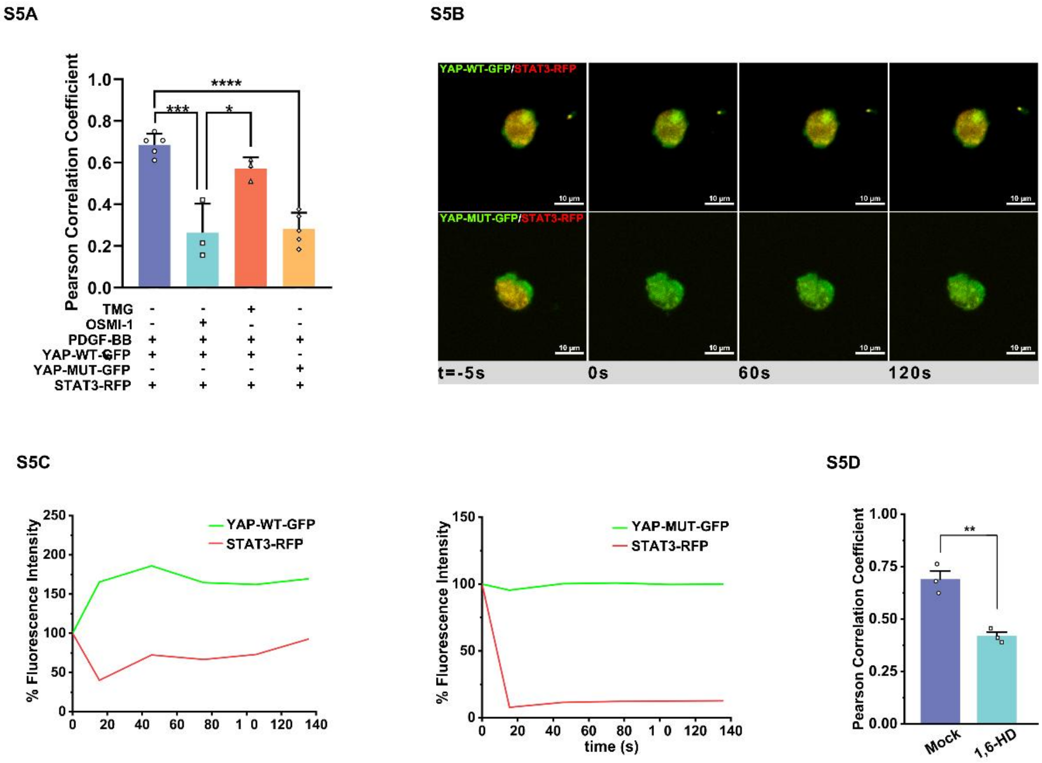

Figure S5. (A) Pearson correlation coefficient between YAP and STAT3 in cytoplasm in Figure 5E (n = 3). * = p<0.05, *** = p<0.005, **** = p<0.001 (unpaired, two-tailed student's test). (B) Co-transfection of PMASMCs with STAT3-RFP and either YAP-WT-GFP or YAP-MUT-GFP plasmids for 48 hours. Fluorescence bleaching in the RFP wavelength band at time 0, and observation of GFP and RFP fluorescence intensities at 60 and 120 seconds. (C) Graphical representation of the relative fluorescence intensity of (B) over time. (D) Pearson correlation coefficient between YAP and STAT3 in cytoplasm in Figure 5F (n = 3). ** = p < 0.01 (unpaired, two-tailed student's test).

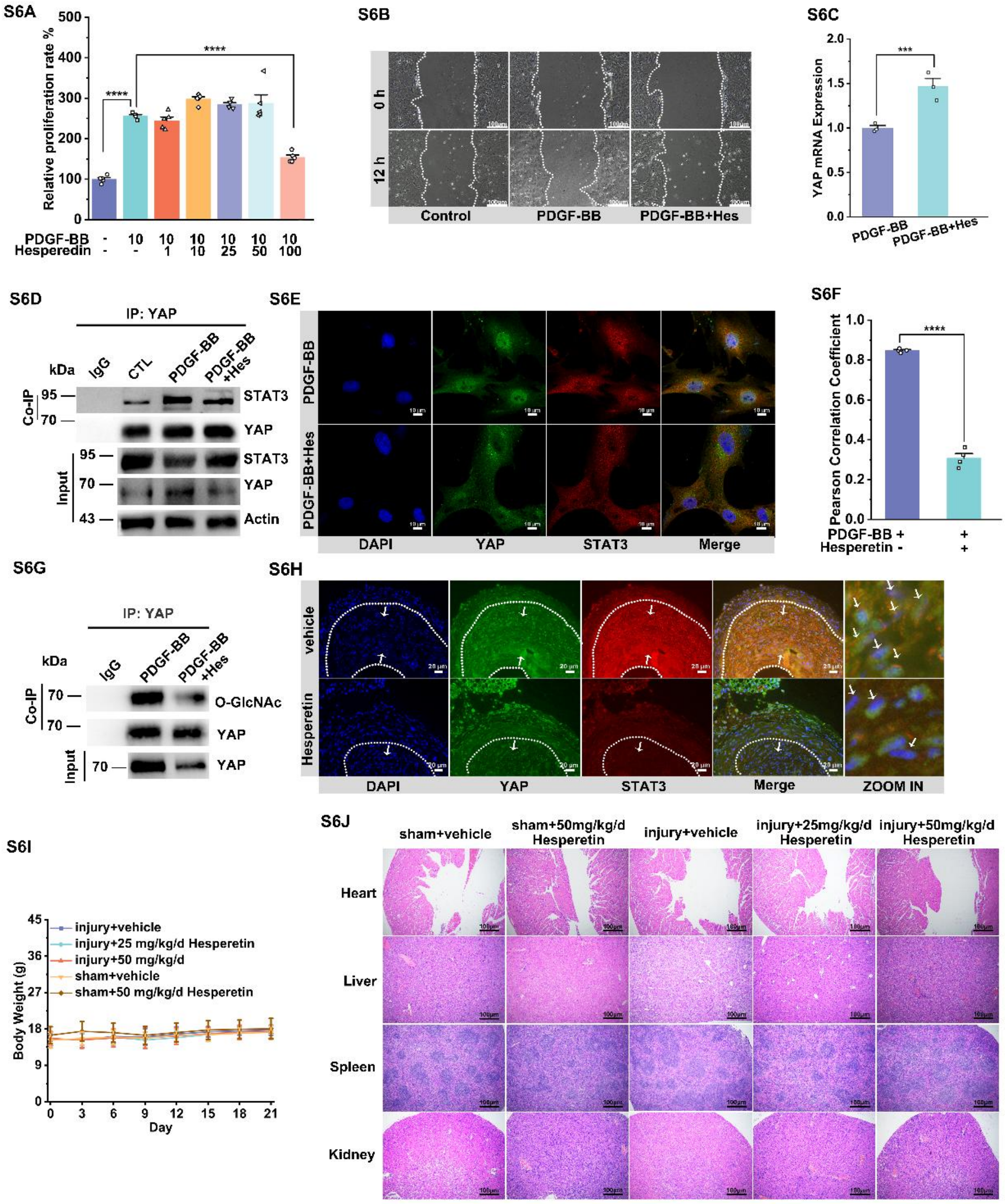

Figure S6. (A) CCK8 assay measured OD value at 450 nm of PMASMCs were treated with PDGF-BB (10 ng/mL) and different concentrations of Hesperidin (0, 1, 10, 25, 50, 100μM), respectively, for 24 h (n = 5). Data are mean ± SEM. **** = p < 0.001 (unpaired, two-tailed student's test). ((B-G) PMASMCs were treated with PDGF-BB (10 ng/mL) and Hesperidin (100μM). (B) Wound healing assay showed the migration rate of cells (n = 3). (C) mRNA expression of CYR61(n = 3), Data are mean ± SEM,

*** = $p < 0.005$ (one-way ANOVA with Tukey's posttest). (D) Immunoblot analysis of the interaction between endogenous YAP and STAT3 in cells (n = 3). (E) Confocal imaging and (F) Pearson correlation coefficient between YAP (green) and STAT3 (red) co-localization in cells (n = 3). **** = $p < 0.001$ (unpaired, two-tailed student's test). (G) Immunoblot analysis of O-GlcNAcylated YAP in cells using an O-GlcNAc-specific antibody (n = 3). (H-J) Mice subjected to carotid artery ligation and oral administration of corn oil (Vehicle group) or hesperidin (50 mg/kg/d) for 21 days. (H) Immunofluorescence of paraffin sections showed neointima formation and YAP-STAT3 co-localization, scale bar, 20μm. (I) Body weight data of carotid artery ligated mice since the addition of hesperidin for 21 d, n = 11. (J) HE for safety of carotid ligation in mice with nerolidol added at 21 d. scale bar, 100μm.
